# Hypoxia-Driven HIF-1α Activation Reprograms Pre-Activated NK Cells towards Highly Potent Effector Phenotypes via ERK/STAT3 Pathways

**DOI:** 10.3390/cancers13081904

**Published:** 2021-04-15

**Authors:** Seon Ah Lim, Yunwon Moon, Min Hwa Shin, Tae-Jin Kim, Sehyun Chae, Cassian Yee, Daehee Hwang, Hyunsung Park, Kyung-Mi Lee

**Affiliations:** 1 Department of Biochemistry and Molecular Biology, College of Medicine, Korea University, Seoul 02841, Korea; reallysun@gmail.com (S.A.L.); mhshin0423@gmail.com (M.H.S.); anaros33@gmail.com (T.-J.K.); 2 Department of Life Science, University of Seoul, Seoul 02504, Korea; wind520@hanmail.net; 3 Korea Brain Bank, Korea Brain Research Institute, Daegu 41068, Korea; implish@postech.ac.kr; 4 Departments of Melanoma Medical Oncology and Immunology, MD Anderson Cancer Center, Houston, TX 77054, USA; cyee@mdanderson.org; 5 School of Biological Sciences, Seoul National University, Seoul 08826, Korea; daehee@snu.ac.kr; 6 Department of Biomedical Engineering, Center for Bio-Integrated Electronics, Simpson Querrey Institute, Northwestern University, Evanston, IL 60208, USA

**Keywords:** NK cells, hypoxia, HIF-1/ERK/STAT3, tumor microenvironment

## Abstract

**Simple Summary:**

In patients with advanced cancer, hypoxic stress shapes NK cells toward tumor-resistant and immunosuppressive phenotypes. Therefore, a strategy to restore NK cell function within hypoxia would be crucial for successful tumor immunotherapy. By manipulating pO_2_ exposure to naïve vs. pre-activated NK cells, we found that HIF-1α-dependent metabolic reprogramming of NK cells is the key to overcoming hypoxia-mediated NK cell impairment. Exposure of pre-activated NK cells to hypoxia with 1.5% pO_2_ initiated metabolic shift from oxidative phosphorylation to glycolysis and reduction of p21/p53-dependent apoptotic pathways, with concomitant upregulation of cell cycle-promoting genes and downregulation of cell cycle-arrest genes via HIF-1a/ERK/STAT3 activation. Furthermore, upregulation of NKp44 activating receptor in hypoxia-exposed pre-activated NK cells elevated cytotoxicity of K562, CEM, and A375 tumor cells, in both in-vitro and in-vivo tumor-clearance assays. Therefore, HIF-1α-mediated metabolic reprogramming of NK cells could reverse their impaired phenotype, generating functionally robust NK cells for adoptive therapy and clinical evaluation.

**Abstract:**

NK cells are the predominant innate lymphocyte subsets specialized to kill malignant tumor cells. In patients with advanced cancer, hypoxic stress shapes NK cells toward tumor-resistant and immunosuppressive phenotypes, hence a strategy to restore NK function is critical for successful tumor immunotherapy. Here, we present evidence that pre-activation and subsequent HIF-1α-dependent metabolic shift of NK cells from oxidative phosphorylation into glycolysis are keys to overcome hypoxia-mediated impairment in NK cell survival, proliferation, and tumor cytotoxicity. Specifically, exposing NK cells to 7–9 days of normoxic culture followed by a pO_2_ of 1.5% hypoxia led to a highly potent effector phenotype via HIF-1α stabilization and upregulation of its target genes, *BNIP3*, *PDK1*, *VEGF*, *PKM2*, and *LDHA*. RNA sequencing and network analyses revealed that concomitant reduction of p21/p53 apoptotic pathways along with upregulation of cell cycle-promoting genes, *CCNE1*, *CDC6*, *CDC20,* and downregulation of cell cycle-arrest genes, *CDKN1A*, *GADD45A*, and *MDM2* were accountable for superior expansion of NK cells via ERK/STAT3 activation. Furthermore, HIF-1α-dependent upregulation of the NKp44 receptor in hypoxia-exposed NK cells resulted in increased killing against K562, CEM, and A375 tumor targets both in-vitro and in-vivo tumor clearance assays. Therefore, hypoxic exposure on pre-activated proliferating NK cells triggered HIF-1α-dependent pathways to initiate coordinated regulation of cell cycle, apoptosis, and cytotoxicity at the global gene transcription level. Our results uncover a previously unidentified role of HIF-1α-mediated metabolic reprogramming that can reverse impaired NK effector phenotypes to generate requisite numbers of functionally robust NK cells for adoptive cellular therapy for clinical evaluation.

## 1. Introduction

As a part of innate immunity, natural killer (NK) cells play a major role in the lysis of tumors and virus-infected cells. Their lytic functions are regulated by an array of activating and inhibitory receptors on the cell surface, leading to the release of small granules containing perforin and granzymes [1,2]. Activated NK cells are cytotoxic to tumor cells and produce immunomodulatory cytokines, such as interferon (IFN)-γ and tumor necrosis factor (TNF)-α [3]. IFN-γ is a key effector cytokine secreted by NK cells and plays a major role in antitumor activity. NK cell activating receptor NKG2D and interleukin (IL)-12 induced by macrophages and neutrophils has been reported to produce IFN-γ, and this can be enhanced by TNF-α [4,5]. Recent report shows that growth factor such as platelet-derived growth factor (PDGF)-DD produced by tumor stimulates NK cells to secrete IFN-γ, TNF-α and chemokines by recognizing NKp44 [6]. NK cell cytokines secreted by PDGF-DD-NKp44 interaction promote the decrease of tumor cell-cycle regulating genes and tumor growth arrest [6]. Despite their effective anti-tumor immunity, NK cell function is largely impaired by the immunosuppressive tumor microenvironment due to multiple cellular and metabolic factors produced by tumor cells under hypoxia [7,8,9]. Hypoxic stress within the tumor microenvironment shapes the NK cell phenotype, thereby inducing tumor resistance and generating immune-suppressive cells. For example, exposure of NK cells to hypoxic conditions of 1% pO_2_ resulted in drastic suppression of NK cytotoxicity against natural tumor targets [10,11,12]. Furthermore, tumor cells themselves adopt several strategies to evade NK cell-mediated killing by directly downregulating NK-activating receptors and/or indirectly releasing immunosuppressive factors such as TGFβ, IL-10, IDO, and soluble NK receptor ligands, namely ULBPs and MICA [12]. Therefore, development of a therapeutic strategy to restore impaired NK function (which could be relevant to patients with advanced stages of tumor) would be crucial for successful tumor immunotherapy.

Upon normoxic-to-hypoxic switch during ex-vivo culture, an array of gene transcriptional events is triggered [13,14]. A transcription factor named hypoxia-inducible factor (HIF), whose activity is regulated by oxygen-dependent degradation, becomes stabilized immediately after hypoxic exposure and forms a heterodimer with a nuclear protein HIF-1β/Arnt. The heterodimeric HIF-1α/β binds to the hypoxia-responsive elements to initiate the transcription of downstream target genes including *BNIP3*, *LDHA*, *PKM2*, *EPO*, and *VEGF* [15], thus triggering tumor progression, dissemination, metastasis, and chemoresistance in multiple cancers such as colorectal cancer, breast cancer, cervical cancer, glioma, and pancreatic cancer [16,17,18,19,20]. Hypoxic induction of many glycolytic enzymes such as PKM2 and LDHA and negative regulators of mitochondrial function such as PDK1 and BNIP3 facilitates the metabolic shift from oxidative phosphorylation to glycolysis, which is a feature of stem cells [21] and long-lived NK cells [22]. PDK1 is known to inactivate pyruvate dehydrogenase (PDH), which converts pyruvate to acetyl-CoA, leading to the shutdown of TCA cycle in mitochondria [23]. BNIP3 also reduces mitochondrial function by inducing mitophagy [24]. Deletion of HIF-1α impairs cell-cycle progression and induces apoptosis in leukemia cells via upregulation of p16 and p19 apoptosis-related genes [25]. In humans, upregulation of HIF-1α affects NK cell function rather negatively by downregulating the expression of activating receptors involved in tumor killing [10]. Notably, specific deletion of HIF-1α in NK cells impairs NK cell tumor cytotoxicity [26], indicating that HIF-1α is also essential for maintaining tumor immunosurveillance in NK cells, besides its function as a hypoxia-responsive transcription factor. Therefore, how hypoxic environment and/or HIF-1α-induced transcriptional events lead to changes in NK cell metabolic processes and cytolytic function against tumor cells, within the tumor microenvironment, still remain unclear.

Recent studies have reported hypoxia-induced impairment of NK cell tumor cytotoxicity against multiple myeloma to be restored by pre-activation of IL-2 ex vivo [11,27]. Furthermore, memory-like properties of NK cells, with elevated cytotoxicity, can be obtained upon repeated exposure to either viral infections in vivo [28,29] or combined pre-activation of IL-12, IL-15, and IL-18 cytokines ex vivo [27]. Intrigued by these findings, we hypothesized that pre-activation of NK cells, prior to hypoxic switch, is the key to initiating hypoxia- or HIF-1α-induced signaling events toward the beneficial gain of function, leading to elevated cytotoxicity with increased cell expansion, as seen in memory-like NK cells. Here, we demonstrated that optimal ex-vivo culture time and O_2_ concentration can indeed lead to metabolic reprogramming of NK cells and induce a potent effector phenotype to overcome hypoxia-induced NK cell impairment via activation of the HIF-1α/ERK/STAT3 pathways.

## 2. Results

### 2.1. Exposure of NK Cells to Hypoxia Impairs Their Proliferation and Tumor Cytotoxicity

**Figure 1:**
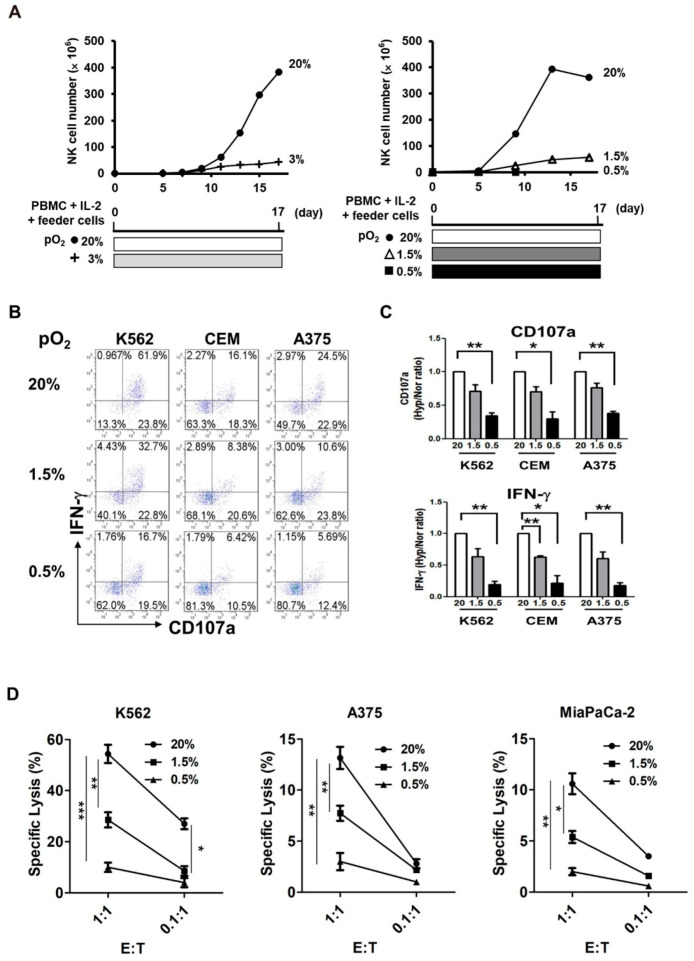
Hypoxic exposure of resting NK cells impairs their proliferation and anti-tumor activities ex-vivo. (**A**) Time course of NK cell expansion under different pO_2_ conditions. PBMCs were isolated from peripheral blood and co-cultured with irradiated KL-1 and LCL feeder cell lines at a ratio of 1:0.5:0.5 in presence of 500 U/mL IL-2 under different pO_2_ conditions (20, 3, 1.5, and 0.5%) for 21 days. Due to limited availability of hypoxic chamber, 3 and 1.5% hypoxia experiments were performed separately. (**B**) Representative FACS plots (*n* = 3) demonstrating CD107a expression and intracellular IFN-γ content of NK cells cultured under different pO_2_ conditions. PBMCs were exposed to different pO_2_ conditions (20, 1.5, and 0.5%) for 96 h, along with feeder cells and IL-2, and CD107a degranulation and IFN-γ production by NK cells were measured by FACS after incubating the PBMCs with target tumor cells under normoxic condition for 6 h. (**C**) The ratios of CD107a+ or IFN-γ+ NK cells in normoxic vs. hypoxic condition from three independent experiments are plotted as bar graphs. (**D**) NK cell tumor cytotoxicity observed after 96-h incubation with target cells under different pO_2_ conditions (20, 1.5, and 0.5%) was determined by LDH cytotoxicity detection kit. LDH activity released from the cytosol of damaged tumor cells was measured after incubating purified NK cells with target cells under normoxic condition for 4 h. The percentage of specific lysis was calculated as follows: [((abs 492 nm effector/target mix − abs 492 nm effector only) − abs 492 nm spontaneous)/(abs 492 nm maximum − abs 492 nm spontaneous)] × 100. Data are presented as the mean ± SEM from three independent experiments. *, *p* < 0.05; **, *p* < 0.01; ***, *p* < 0.001.

To investigate the effect of hypoxia on NK cell proliferation, whole PBMCs were cultured under 0.5, 1.5, or 3% pO_2_ in presence of irradiated feeder cell lines KL-1 and lymphoblastoid B cell line (LCL), which are known to induce NK cell enrichment and proliferation [30]. While NK cells in 20% pO_2_ reached up to 400-fold expansion by day 17, those cultured in 3 or 1.5% pO_2_ proliferated only up to 20-fold and those cultured in 0.5% pO_2_ failed to proliferate at all (Figure 1A). Next, we examined the effect of hypoxia on tumor cytotoxicity and cytokine secretion in NK cells. After 96-h exposure of PBMCs to hypoxia (1.5 or 0.5% pO_2_ ex vivo), we measured CD107a degranulation and IFN-γ production by NK cells against three different tumor targets—a human leukemia cell line K562, a human lymphoblastoid cell line CEM, and a human melanoma cell line A375—using fluorescence-activated cell sorting (FACS). The percentage of CD107a+ NK cells, against all three tumor targets, decreased in a pO_2_-dependent manner (Figure 1B; pO_2_ of 20, 1.5, and 0.5% corresponded to 85.7, 55.5, and 36.2%, respectively, for K562; 34.4, 28.98, and 16.92%, respectively, for CEM; and 47.4, 34.4, and 18.09%, respectively, for A375). Similarly, the percentage of IFN-γ+ NK cells also decreased in a pO_2_-dependent manner (Figure 1B; pO_2_ of 20, 1.5, and 0.5% corresponded to 62.9, 37.1, and 18.5%, respectively, for K562; 18.4, 11.3, and 8.2%, respectively, for CEM; and 27.5, 13.6, and 6.8%, respectively, for A375). The mean ratios of CD107a+ and IFN-γ+ NK cells, under normoxic vs. hypoxic conditions, are summarized in Figure 1C. NK cell tumor cytotoxicity measured by LDH cytotoxicity kit was also decreased in a pO_2_-dependent manner, indicating that hypoxia inhibited anti-tumor activity of NK cells against all three tumor targets, K562, A375, and MiaPaCa-2, a pancreatic tumor cell line (Figure 1D). Overall, the data implied that circulating NK cells that reached the hypoxic tumor mass could not perform efficient tumor cytolysis and improve IFN-γ production. Furthermore, their proliferation was greatly impaired under the hypoxic microenvironment.

### 2.2. Pre-Activation of PBMCs Prior to Hypoxic Exposure Increases Ex-Vivo NK Cell Expansion

**Figure 2:**
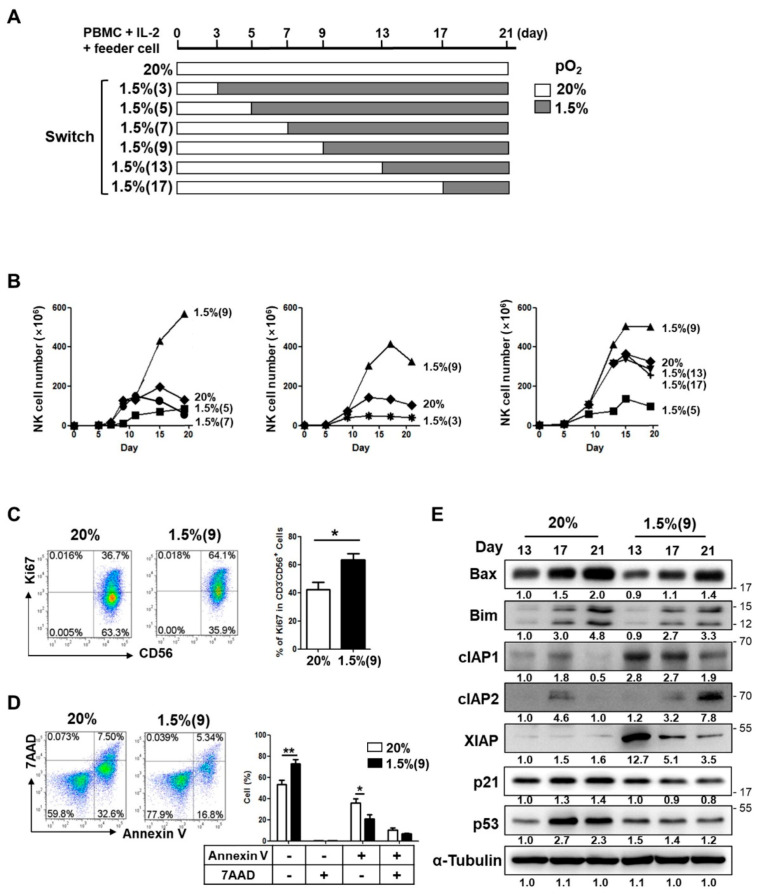
Time-controlled exposure of NK cells to hypoxia can lead to a highly proliferative phenotype. (**A**) Schematic representation, showing the dates of normoxia-to-hypoxia switch, during NK cell culture ex-vivo. (**B**) Representative cell counting results over the course of 21-day culture, under normoxia and hypoxia, are shown. (**C**) **Left**, rate of proliferation of NK cells in normoxic and hypoxic cultures was assessed by Ki67 staining and analyzed by flow cytometry. **Right**, the levels of Ki67^+^ population, shown in left panel, from six independent experiments are plotted as bar graphs (mean ± SEM *, *p* < 0.05; **, *p* < 0.01). (**D**) **Left**, normoxic and hypoxic cultures of NK cells were stained with annexin V/7AAD and analyzed by flow cytometry. Number in each quadrant indicates the percentage of annexin V^+^ and/or 7AAD^+^ population. **Right**, levels of annexin V^+^ and/or 7AAD^+^ population, shown in left, from six independent experiments are plotted as bar graphs. Data are presented as the mean ± SEM. *, *p* < 0.05; **, *p* < 0.01. (**E**) Western blot analyses were performed to assess time-dependent changes of pro-apoptotic (Bax and Bim), anti-apoptotic (cIAP1, cIAP2, and XIAP), and cell cycle arrest (p21 and p53) marker expression in normoxic and hypoxic cultures of NK cells. Density of each band was normalized to that of α-tubulin. Data shown are representative of three independent experiments.

Since NK cell populations within the hypoxic tumor microenvironment may be heterogeneous, depending on their activation status, we aimed to explore whether the activated NK cells could lose their proliferative potential under hypoxic conditions, similar to resting NK cells. To test this, we first activated NK cells under normoxia (20% pO_2_) in the presence of IL-2 and feeder cells, for various durations (3, 5, 7, 9, 13, 17, and 21 days) prior to hypoxic exposure (Figure 2A). NK cells exposed to 1.5% pO_2_ (hypoxia) after normoxic culture for 3, 5, or 7 days showed lesser proliferation than those not exposed to hypoxia at all (Figure 2B, left and middle panel). In contrast, NK cell proliferation upon normoxic-to-hypoxic switch after 9 days of culture showed a significantly greater increase of up to 4.3-fold (***, *p* < 0.001), compared to that in the absence of hypoxia. No further increase in NK cell expansion was observed when the hypoxic switch occurred at 13 or 17 days (Figure 2B, right panel). These data suggested that enhanced proliferation of NK cells due to IL-2 pre-activation is evident only for hypoxic switches occurring within a specific time window. All replicates of NK cell expansion graphs, originating from three to eight separate donors, are shown in Supplementary Figure S1.

On day 9 of hypoxic culture of the pre-activated NK cells, we evaluated the rates of cell proliferation and apoptosis by measuring the percentage of NK cells expressing Ki67 and annexin V/7AAD, respectively. The hypoxic switch significantly increased (*p* < 0.05) the number of proliferating Ki67^+^CD3^−^CD56^+^ NK cells from 36.7% to 64.1% (Figure 2C). In contrast, it decreased the percentage of apoptotic NK cells expressing annexin V^+^/7AAD^−^ (early apoptosis; 32.6% to 16.8%) and annexin V^+^/7AAD^+^ (late apoptosis; 7.5% to 5.34%) (Figure 2D). In accordance with these findings, the normoxic-to-hypoxic switch in the pre-activated NK cells was found to drastically reduce the levels of pro-apoptotic Bax and Bim proteins and increase the levels of anti-apoptotic cIAP1, cIAP2, and XIAP proteins during the culture up to 21 days (Figure 2E, Supplementary Figure S4). More importantly, the levels of p21 and p53 tumor suppressors were found to be significantly lower in the NK cells that underwent normoxic-to-hypoxic switch.

Since the NK cells used in this study were obtained from expanded PBMCs stimulated with feeder cells and IL-2, one could argue that the enhanced NK cell proliferation and effector function seen in the normoxic-to-hypoxic culture condition might have been due to the presence of non-NK cells in the PBMC cultures. To test this possibility, we purified NK cells up to 95% purity on day 9 of normoxic culture and exposed the pure NK cells to hypoxic conditions (1.5% pO_2_). Similar to our initial results, the highly purified NK cells showed enhanced proliferation upon switching to hypoxia (1.5% pO_2_), as shown in Supplementary Figure S2. Western blot results of NK cells cultured under normoxic-to-hypoxic conditions [1.5%(9)] demonstrated downregulated pro-apoptotic Bim protein and upregulated anti-apoptotic cIAP1, cIAP2, and XIAP proteins as compared to those cultured in normoxic conditions (20%) at any given time points of culture (days 13, 17, 21)

Taken together, the increased expansion of pre-activated NK cells, following normoxic-to-hypoxic switch, seemed to be primarily due to accelerated cell cycle progression along with suppressed apoptotic pathways owing to direct hypoxic influence on NK cells.

### 2.3. Pre-Activated NK Cells Show Downregulation of Genes Involved in Cell Cycle Arrest and Cell Death When Exposed to Hypoxia

**Figure 3:**
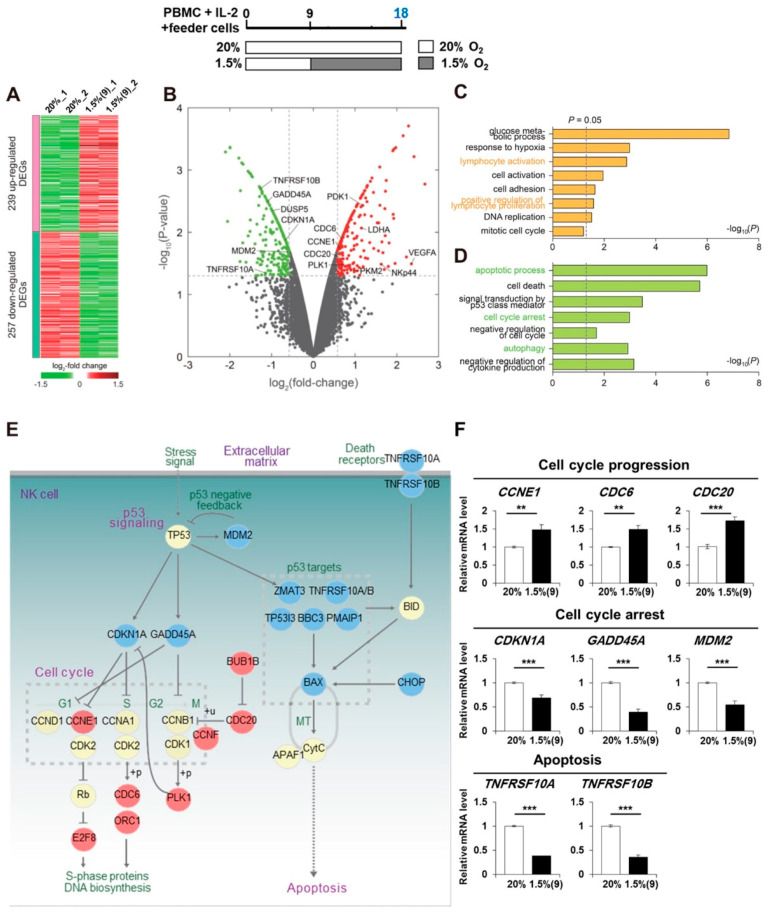
Pre-activated hypoxic NK cells upregulate cell cycle and downregulate cell-cycle arrest and apoptosis. (**A**) Heat map shows upregulated and downregulated genes in pre-activated hypoxic NK cells, compared to those in normoxic NK cells. RNA was isolated from purified NK cells in each condition, as described in Materials and Methods, for processing RNA-seq. RNA-seq analyses were performed using the two samples from two donors. Red and green represent upregulated and downregulated genes, respectively. (**B**) Volcano plot showing more differentially expressed genes in the pre-activated hypoxic NK cells than in the normoxic NK cells. Red and green dots represent upregulated and downregulated genes, respectively, and gray dots represent genes whose expression was not significantly different. (**C**,**D**) GOBPs enriched by the upregulated (**C**) and downregulated (**D**) genes. Enrichment significance is displayed as −log_10_(P), where P is the enrichment *p*-value for the corresponding GOBP. (**E**) The network model, describing the interplays among the upregulated or downregulated genes involved in cell cycle arrest, progression, and apoptosis (Kyoto Encyclopedia of Genes and Genomes pathway database). Node colors represent upregulation (red), downregulation (blue), and no change (yellow) of the corresponding genes in the pre-activated hypoxic NK cells, compared to that in normoxic NK cells. Solid and dotted lines denote direct and indirect interactions, respectively. (**F**) Relative mRNA levels of the denoted genes in normoxic and pre-activated hypoxic NK cells at day 23 were analyzed by quantitative RT-PCR. NK cells in PBMCs were expanded with IL-2 and feeder cells under normoxic or hypoxic conditions and then isolated. mRNA level of each gene was normalized using the 18S rRNA level. Data obtained from more than two independent experiments, each using PBMCs from different individual donors, are presented as the mean ± SEM. **, *p* < 0.01; ***, *p* < 0.001 by Student’s two-tailed *t*-test.

To understand the molecular signatures for enhanced proliferation of NK cells under hypoxia, we performed mRNA-sequencing of the normoxia-cultured and pre-activated/hypoxia-exposed NK cells (abbreviated henceforth as pre-activated hypoxic NK cells). Comparing mRNA expression profiles, we identified 496 differentially expressed genes (DEGs; 239 upregulated and 257 downregulated) between the two differentially cultured NK cells (Figure 3A; Supplementary Table S1). The expression of several genes involved in cell cycle promotion (CCNE and CDC6/20) was upregulated while that of others involved in cell cycle arrest (CDKN1A, GADD45A, and MDM2) and apoptosis (TNFRSF10A and TNFRSF10B) was downregulated (Figure 3B). To further examine the cellular processes associated with DEGs, we performed enrichment analysis of gene ontology biological processes (GOBPs). The upregulated genes were significantly (*p* < 0.05) associated with processes related to NK cell activation and proliferation (lymphocyte activation and positive regulation of lymphocyte proliferation, shown in Figure 3C). The downregulated genes were associated with processes related to cell cycle arrest, apoptosis, cell death, and autophagy (Figure 3D).

To understand the collective effect of upregulated cell proliferation and downregulated apoptosis, we built a network model describing the interactions across genes involved in these processes. The network model (Figure 3E) showed downregulation of genes involved in cell cycle arrest (CDKN1A and GADD45A) and upregulation of those involved in cell cycle progression, such as cyclin (CCNE1 and CCNF), cell-division-cycle genes (CDC6 and CDC20), and other regulators (E2F8, BUB1B, PLK1, and ORC1). Downregulation of p53 target genes (CDKN1A/p21, GADD45A, ZMAT3, TNFRSF10A/TRAILR1, TNFRSF10B/TRAILR2, TP53I3, BBC3, PMAIP1, and BAX), involved in cell cycle arrest and apoptosis, was apparent. qRT-PCR analysis further confirmed the upregulation of CCNE1, CDC6, and CDC20 (which are involved in cell cycle progression) in pre-activated hypoxic NK cells; it also showed the downregulation of CDKN1A, GADD45A, and MDM2 (involved in cell cycle arrest) and TNFRSF10A/B (involved in apoptosis; Figure 3F).

Taken together, these data suggested coordinated regulation of cell cycle and apoptosis at the global level to generate the enhanced proliferative potential of pre-activated hypoxic NK cells. Notably, apoptosis (*p* < 0.01) was more strongly associated with the downregulated genes than was cell cycle proliferation (*p* > 0.05) with the upregulated genes (Figure 3C,D), thereby suggesting that the enhanced NK cell proliferation is primarily mediated by decreased apoptosis, as is consistent with the findings in Figure 2D,E.

### 2.4. HIF-1α Is Important for the Enhanced Proliferation of Pre-Activated Hypoxic NK Cells

**Figure 4:**
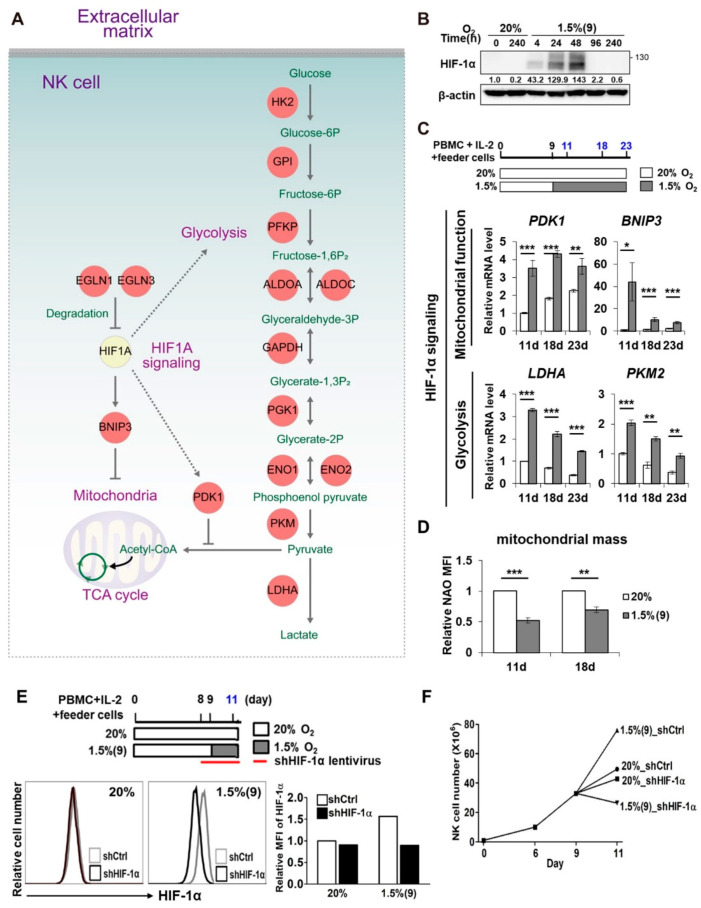
Enhanced proliferation of pre-activated hypoxic NK cells is dependent on HIF-1α activity. (**A**) Network model includes the upregulated HIF-1α signaling and glycolysis. The nodes and lines are as described in Figure 3E. (**B**) Western blot analyses were performed to assess time-dependent changes of HIF-1α expression in normoxic and hypoxic cultured NK cells. β-actin was taken as a loading control. (**C**) qRT-PCR analyses of PDK1, BNIP3, LDHA, and PKM2 in NK cells at days 11, 18, and 23 are shown. NK cells in PBMCs were expanded with IL-2 and feeder cells under normoxic or hypoxic conditions and then isolated on the indicated days. Their expression levels were normalized using that of 18S rRNA and further divided by normalized level of the gene in normoxic NK cells at day 11. Data obtained from more than two independent experiments, each using PBMCs from different individual donors, are presented as the mean ± SEM. *, *p* < 0.05; **, *p* < 0.01; ***, *p* < 0.001. (**D**) Mitochondrial mass in NK cells at days 11 and 18 was assessed by NAO staining and analyzed by flow cytometry. Relative levels of mean fluorescence intensity (MFI) from five independent experiments are plotted as bar graphs. Data are presented as the mean ± SEM. **, *p* < 0.01; ***, *p* < 0.001. (**E**) **Upper**, schematic representation of HIF-1α knockdown. NK cells were infected with lentivirus containing shCtrl and shHIF-1α at day 8 and exposed to pO_2_ of 1.5% after 9-day expansion in normoxia. **Lower**, HIF-1α knockdown was verified using intracellular HIF-1α flow cytometry (left) and HIF-1α mean fluorescence intensity (MFI) (right). (**F**) Time course of ex-vivo expansion of shCtrl or shHIF-1α NK cells.

HIF-1α is a well-known transcription factor that is elevated at the protein level upon hypoxic exposure, although its mRNA level often shows no change [15,31]. Immediate metabolic changes that occur upon hypoxic exposure and HIF-1α activation include the upregulation of genes involved in glycolysis [31,32]. Consistent with this observation, we found glycolysis to be highly enriched considering the upregulation of related genes (Figure 3C). The network model also revealed the upregulation of 11 genes involved in glycolysis, along with other well-known target genes of HIF-1α, such as PDK1 and BNIP3 (Figure 4A). These data highlighted the activation of HIF-1α in pre-activated hypoxic NK cells. Indeed, HIF-1α protein levels were significantly increased in pre-activated hypoxic NK cells for up to 48 h and reduced to basal levels after 96 h of exposure to hypoxia, as shown in the western blots using anti-HIF-1α antibody (Figure 4B, Supplementary Figure S4). We also measured HIF-2α level (another HIF-α isoform) and found its protein level to not be significantly altered in the pre-activated hypoxic NK cells (Supplementary Figure S3). Next, we examined whether such an early and transient increase in HIF-1α could be sufficient to induce its target genes after a normoxic-to-hypoxic switch. qRT-PCR analysis revealed the HIF-1α target genes to be upregulated within 48 h post hypoxic exposure and to gradually decline after 18 days (Figure 4C), consistent with the transient induction pattern of HIF-1α. Mitochondrial mass in pre-activated hypoxic NK cells was found to be lower than that in NK cells cultured under normoxia (Figure 4D). Next, we generated HIF-1α-knockdown NK cells by infecting NK cells with lentivirus encoding HIF-1α shRNA and evaluated the effect on proliferation of NK cells. We first confirmed the knockdown of HIF-1α using flow cytometry to detect intracellular stained HIF-1α. HIF-1α flow cytometry panels and mean fluorescence intensity (MFI) showed decreased HIF-1α protein levels at 48 h (11 days), following the hypoxic switch on day 9 (Figure 4E). Notably, HIF-1α knockdown reduced the proliferation of pre-activated hypoxic NK cells to a greater extent than that of normoxic NK cells (Figure 4F). Taken together, these data demonstrated HIF-1α activation to be critical for the enhanced proliferation of pre-activated hypoxic NK cells.

### 2.5. Enhanced Proliferation of Pre-Activated Hypoxic NK Cells Is Mediated by ERK and STAT3 Signaling Pathways

**Figure 5:**
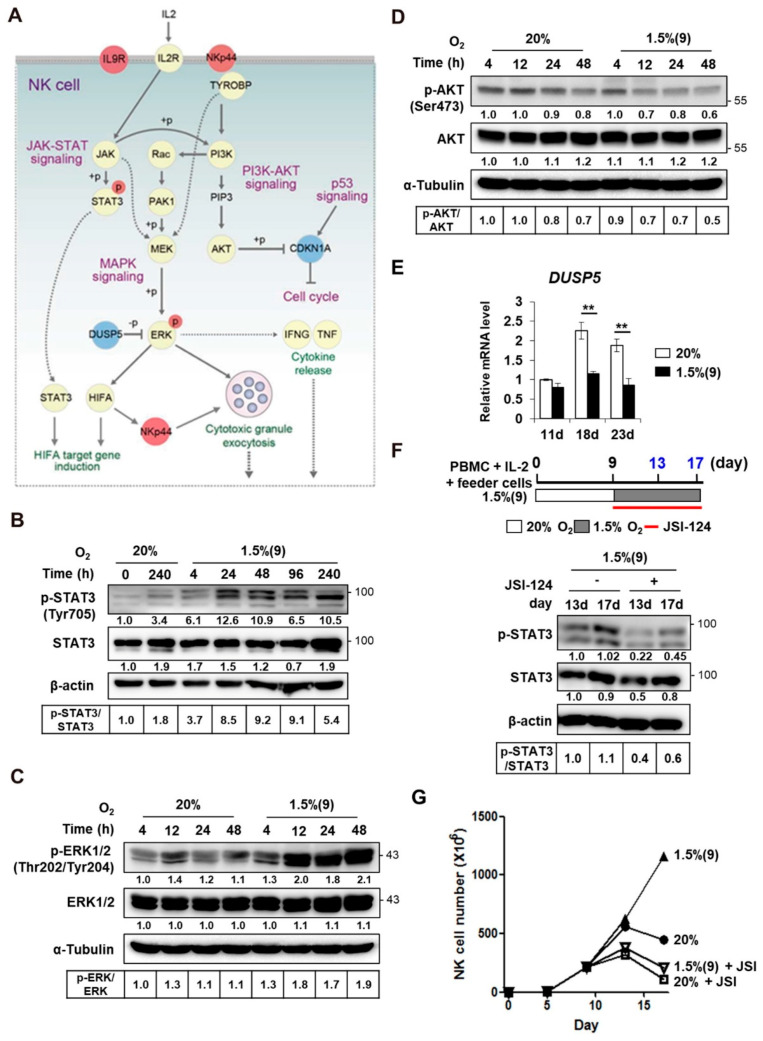
Enhanced proliferation of pre-activated hypoxic NK cells is related to ERK and STAT3 activity. (**A**) Network model includes interactions among the three major signaling pathways, STAT, ERK, and PI3K-AKT. The nodes and lines are as described in Figure 3E. Western blot analyses were performed to assess time-dependent changes of STAT3 (**B**), ERK (**C**), and AKT (**D**) phosphorylation in normoxic and hypoxic cultures of NK cells, following 9 days of culture in normoxia. Fold activation was calculated using densitometry, with β-actin or α-tubulin as a reference and is depicted under each band. The density of phospho-protein/total protein was calculated and provided below the band. (**E**) qRT-PCR analyses of DUSP5; NK cells in PBMCs were expanded with IL-2 and feeder cells under normoxic or hypoxic conditions and then isolated at the indicated days. The mRNA level of DUSP5 was normalized with respect to that of 18S rRNA. Data obtained from two independent experiments, each using PBMCs from different individual donors, are presented as the mean ± SEM. **, *p* < 0.01. (**F**) **Upper**, schematic representation, showing the treatment of STAT3 inhibitor JSI-124. NK cells were exposed to pO_2_ of 1.5% in presence of 0.05 μM JSI-124 after 9-day expansion in normoxia. **Bottom**, levels of p-STAT3 and STAT3 in normoxic and pre-activated hypoxic NK cells, in presence or absence of JSI-124 for 4 or 8 days (at 13 or 17 days). (**G**) The number of NK cells, cultured under normoxic or hypoxic conditions and treated with JSI-124, is plotted against time.

The network model describing the downstream pathways of IL-2 receptor signaling showed that IL-2 pre-activation could lead to the activation of JAK-STAT, MAPK, and PI3K-AKT signaling in pre-activated hypoxic NK cells (Figure 5A). Thus, we aimed to examine whether these downstream signaling pathways were indeed activated in the pre-activated hypoxic NK cells, relative to those in normoxic NK cells. As shown in a previous report, ERK signaling and hypoxic signaling activate each other mutually [33]. In this study, STAT3 and ERK showed increased phosphorylation in pre-activated hypoxic NK cells (Figure 5B,C, Supplementary Figure S4), whereas AKT showed no change in phosphorylation (Figure 5D, Supplementary Figure S4). Downregulated expression of DUSP5, an ERK-specific phosphatase, might also have contributed to the increased ERK signaling in hypoxia (Figure 5E) [34]. Since transactivity of HIF-1α can also be triggered by ERK signaling, contribution of the latter to not only proliferation but also HIF-1α activation would be quite expected. Consistent with the network model (Figure 5A), STAT3 and HIF-1α have been known to cooperatively regulate the induction of HIF-1α target genes [35]. To determine the role of STAT3 signaling in the proliferation of NK cells, we treated pre-activated hypoxic NK cells with JSI-124, a STAT3 inhibitor [36,37]. JSI-124 treatment reduced the level of STAT3 phosphorylation (Figure 5F, Supplementary Figure S4) and significantly decreased the proliferation of pre-activated hypoxic NK cells to less than that of normoxic NK cells (Figure 5G). Taken together, our data suggested that the enhanced proliferation of pre-activated hypoxic NK cells is mediated primarily by the STAT3 and ERK signaling pathways.

### 2.6. Pre-Activated Hypoxic NK Cells Show Enhanced Cytotoxicity after Hypoxic Switch

**Figure 6:**
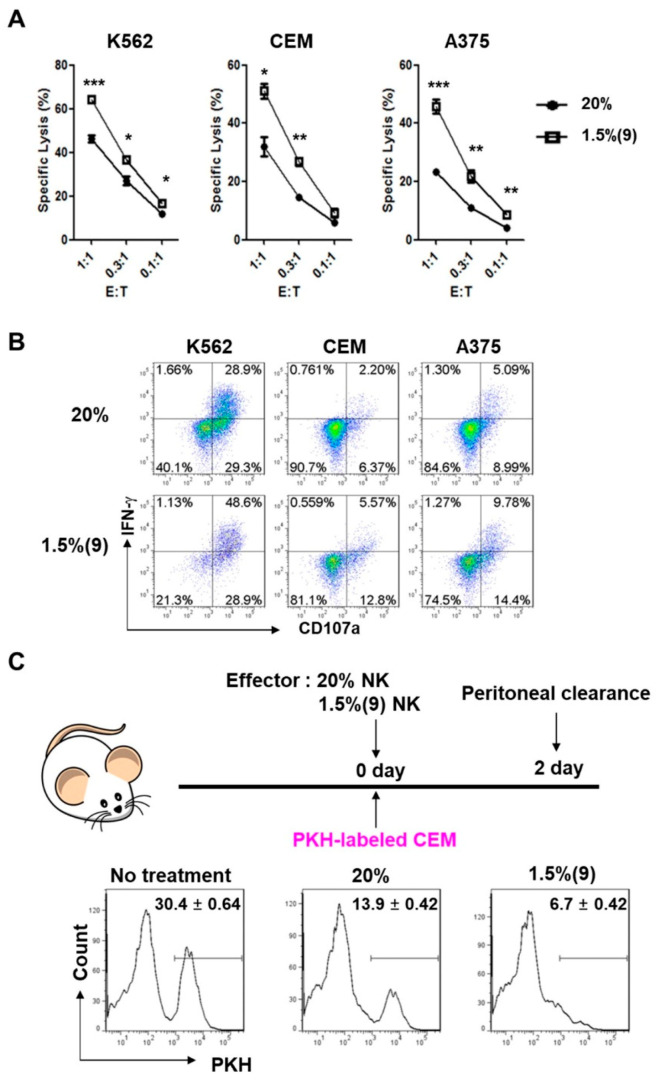
Pre-activated hypoxic NK cells show enhanced cytotoxicity, both in vitro and in vivo. (**A**) Normoxic and pre-activated hypoxic NK cells were assessed for their tumor killing activity using a ^51^Cr release assay against K562, A375, and CEM as targets. The data are from three independent experiments, each run “in triplicate”. The percentage of ^51^Cr release is shown as the mean ± SEM of triplicates. *, *p* < 0.05; **, *p* < 0.01; ***, *p* < 0.001. (**B**) The level of degranulation and cytokine production of NK cells, in response to K562, CEM, and A375 tumor targets, was assessed by surface CD107a and intracellular IFN-γ-staining. Ex-vivo expanded NK cells, under 18-day normoxic or hypoxic conditions, were used as effector cells, at E:T of 1:1, against tumor targets. Data are representative of three independent experiments. (**C**) In-vivo cytolytic activity of NK cells is shown. A total of 5 × 10^6^ CEM tumor cells were labeled with PKH and injected i.p. into 7 to 8-week-old female NSG mice, together with 1 × 10^7^ expanded human NK cells (hNK), cultured in both normoxia and hypoxia. After 2 days, the mice were sacrificed, and peritoneal cells were recovered. The three groups of mice received: tumor cells only, tumor cells with normoxic NK cells, or tumor cells with pre-activated hypoxic NK cells. Extent of tumor killing was assessed by comparing the percentage of PKH^+^ cells recovered from peritoneal lavage, with or without NK cell transfer. Data are presented as the mean ± SEM.

The network model in Figure 5A suggested that activation of ERK signaling can induce cytotoxic granule exocytosis in NK cells. Therefore, we assessed whether pre-activated hypoxic NK cells could show enhanced anti-tumor activity. The results of ^51^Cr release assay revealed the hypoxic switch to significantly (*p* < 0.05) increase the anti-tumor activity of NK cells against all three tumor cell targets, namely K562, CEM, and A375 (Figure 6A). Next, we examined whether the enhanced anti-tumor activity could be associated with NK cell degranulation or IFN-γ production. For this, we measured the changes in CD107a and IFN-γ levels against the three tumor cell lines following the hypoxic switch. The pre-activated hypoxic NK cells showed increased CD107a levels from 58.2 to 77.5% against K562, 8.6 to 18.4% against CEM, and 14.1 to 24.2% against A375 (Figure 6B). Similarly, IFN-γ levels of pre-activated hypoxic NK cells increased from 30.6 to 49.7% against K562, 2.9 to 6.1% against CEM, and 6.4 to 11.1% against A375 (Figure 6B).

To further confirm the enhanced anti-tumor activity in pre-activated hypoxic NK cells in vivo, we adopted a peritoneal clearance model, where 5 × 10^6^ PKH-stained live CEM tumor cells and 1 × 10^7^ cultured human NK cells were co-injected intraperitoneally into immunocompromised NSG (NOD-scid IL2rγnull) mice. The extent of in-vivo lysis was assessed by comparing the percentage of tumor cells remaining after 2 days of intraperitoneal injection between the normoxic NK cells and pre-activated hypoxic NK cells [38]. Without co-injection of NK cells, the percentage of CEM cells in the peritoneum was found to be 30.4 ± 0.6%. However, when normoxic or pre-activated hypoxic NK cells were co-injected with the CEM cells, the percentage of tumor cells remaining in the mouse peritoneum was significantly reduced to 13.9 ± 0.42% or 6.7 ± 0.42%, respectively (Figure 6C). The pre-activated hypoxic NK cells showed an approximately 2.1-fold greater increase in tumor killing activity in vivo than the normoxic NK cells. The data collectively suggested that pre-activated hypoxic NK cells have enhanced anti-tumor effector functions, both ex vivo and in vivo.

### 2.7. Pre-Activated Hypoxic NK Cells Exhibit Higher NKp44 Expression Both at the Transcript and Protein Levels

**Figure 7:**
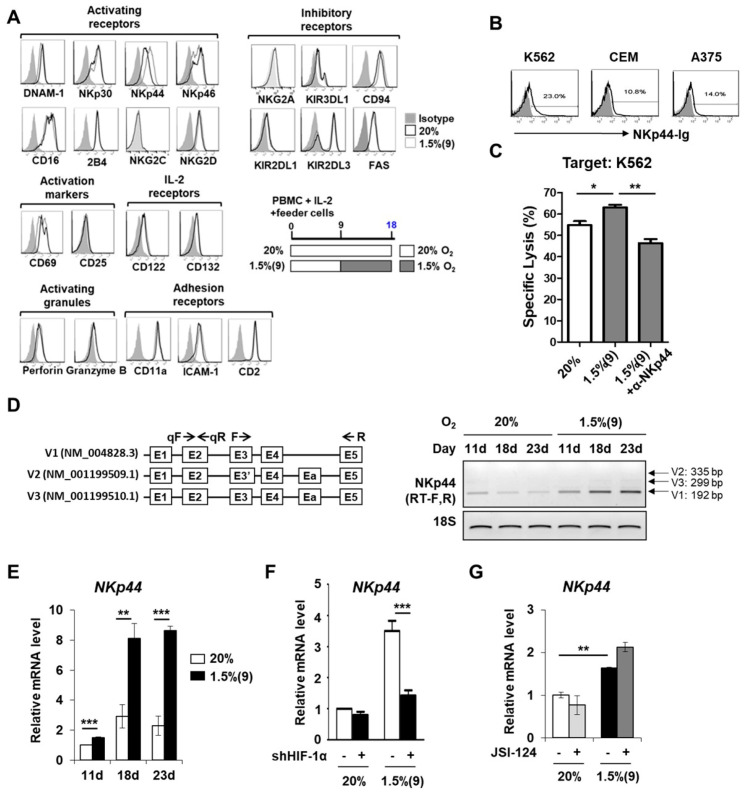
Pre-activated hypoxic NK cells demonstrate high NKp44 expression on the cell surface. (**A**) Surface expression of various receptors was assessed by flow cytometry of NK cells. For each receptor, analysis was performed on CD56^+^CD3^−^ subsets across the whole population. Data shown are representative of four independent experiments. (**B**) Cell surface expression of NKp44L was assessed by flow cytometry. The indicated tumor cell lines were stained with NKp44-Ig fusion protein (black) or controls (gray). (**C**) Tumor killing assays were conducted using ex-vivo expanded NK cells under normoxic or hypoxic conditions for 18 days. Where indicated, pre-activated hypoxic NK cells were treated with blocking anti-NKp44 mAbs at 5 µg/mL during the 4-h cytotoxicity assay. Two experiments were performed, each in triplicate. (**D**) **Left**, diagrams showing three variants of NKp44 mRNA (V1–V3); the primer sets for qRT-PCR and RT-PCR are indicated as qF/qR and F/R respectively, by arrows. **Right**, Expression levels of three NKp44 transcription variants were analyzed by RT-PCR. (**E**) mRNA level of NKp44 was quantified by qRT-PCR. NK cells in PBMCs were expanded with IL-2 and feeder cells under normoxic or hypoxic conditions and then isolated. (**F**) mRNA level of NKp44 in normoxic and pre-activated hypoxic NK cells is shown in presence of shHIF-1α for 2 days (at 11-d). NK cells in PBMCs were expanded with IL-2 and feeder cells under normoxic or hypoxic conditions and enriched up to 85%. (**G**) mRNA level of NKp44 in normoxic and pre-activated hypoxic NK cells, in presence of JSI-124 for 4 days (at 13-d), is shown. NK cells in PBMCs were expanded with IL-2 and feeder cells under normoxic or hypoxic conditions and enriched up to 78%. Data of (**E**–**G**) obtained from two independent experiments, each using PBMCs from different individual donors, are presented as the mean ± SEM. *, *p* < 0.05; **, *p* < 0.01; ***, *p* < 0.001.

We next investigated whether a hypoxia-induced NK cell phenotype, with elevated tumor killing activity, could be associated with changes in NK cell surface receptor profiles. For this, we performed FACS analysis to measure the levels of NK-activating and inhibitory receptors, along with those of perforin and granzyme molecules responsible for cytotoxic granule release (Figure 7A). Compared to normoxic NK cells, pre-activated hypoxic NK cells showed no significant change in the levels of CD11a, ICAM-1, and CD2 adhesion receptors; DNAM-1, NKG2D, NKp30, NKp46, 2B4, and NKG2C activating receptors; CD69 and CD25 activation markers; CD122 and CD132 IL-2 receptors; perforin and granzyme B activating granules; and NKG2A, KIR3DL1, CD94, KIR2DL1, KIR2DL3, and FAS inhibitory receptors. However, expression level of the activating receptor NKp44 was significantly increased (Figure 7A, Supplementary Figure S5). The elevated surface expression of NKp44 in pre-activated hypoxic NK cells correlated with its upregulated gene expression in our network model shown in Figure 5A. Among all the three tumor targets, K562 expressed the surface NKp44 ligands maximally (Figure 7B). Therefore, we examined whether blocking NKp44/NKp44L interactions using anti-NKp44 mAbs during the in-vitro ^51^Cr release assay could affect NK cell activity against K562. As shown in Figure 7C, pre-activated hypoxic NK cells demonstrated greater killing against K562 tumor targets than normoxic NK cells. However, addition of anti-NKp44 mAbs significantly reduced their killing capacity to below that of normoxic NK cells (Figure 7C). The data collectively suggested that upregulation of surface NKp44 in pre-activated hypoxic NK cells is primarily responsible for their enhanced tumor killing activity. Elevated expression of NKp44 is largely associated with its increased mRNA levels, as seen in Figure 7D. Among the three variants of NKp44 (V1–V3) mRNA [39], V1 transcript was mainly upregulated upon hypoxia in pre-activated hypoxic NK cells. The mRNA level of NKp44 gradually increased in NK cells upon hypoxic exposure and reached the maximum level after 14 days of hypoxic switch (Figure 7E). Elevated expression of NKp44 was dependent on HIF-1α activity, rather than on STAT3 activity since knockdown of HIF-1α inhibited the mRNA expression of NKp44 (Figure 7F,G). Collectively, increased tumor killing activity in pre-activated hypoxic NK cells was found to be associated with enhanced NKp44 expression on the cell surface, both at the transcript and protein levels.

## 3. Discussion

Hypoxia promotes the survival of tumor cells while causing immune cell dysfunction within the tumor microenvironment. Exposure of resting NK cells to hypoxia, in this study, resulted in its significantly impaired proliferation ex vivo, suggesting that NK cells reaching the tumor microenvironment would not survive or function. Although NK cells exposed to hypoxia were shown to induce HIF-α stabilization/activation along with its target gene expression [10], similar to those occurring in tumor cells, these hypoxia-mediated signaling events were not associated with the survival benefits of resting NK cells. In this study, we have reported for the first time that pre-activation of NK cells for 9 days, prior to hypoxic exposure, is the key to overcoming the hypoxia-mediated impairments, thereby promoting NK cell survival and proliferation, with enhanced cytolytic activity, via the HIF-1α/ERK/STAT3 pathways. The molecular and transcriptional events between cell cycle and hypoxic signals in human proliferating NK cells could lead to the coordinated regulation of cell cycle, apoptosis, and cytotoxicity at the global transcription level and enable highly functional NK cells even within the hypoxic microenvironment.

Our data showed that NK cells exhibited impaired growth upon hypoxic exposure regardless of the presence of IL-2 in the culture for up to 7 days. However, to our surprise, NK cells exposed to 1.5% hypoxia at day 9, but not day 3, 5, 7, demonstrated robust cell expansion with elevated cytotoxicity. This was quite unexpected, because cells were constantly proliferating following co-culture with IL-2 plus feeder cells in our culture system [30] and nothing was added differently at day 9. Not only in proliferation, but also cytotoxicity was greatly elevated when NK cells were exposed to 1.5% hypoxia at day 9. Therefore, it is not IL-2 that led to the reversal of hypoxia-impaired NK cell expansion and cytotoxicity. Rather the coordinated molecular and transcriptional events occurring at 9 days of NK cell pre-activation were found to be responsible for this enhanced NK proliferation and effector functions. By providing the detailed gene network analysis of NK cells cultured in 20% pO_2_ for 18 days vs. 1.5% pO_2_ exposed for 9 days following 9 days with IL-2 plus feeder cell pre-activation, we present the detailed molecular mechanisms underlying this specific hypoxia-induced synergy in NK cell expansion and tumor cytotoxicity.

The phenotypes presented in pre-activated hypoxic NK cells are quite similar to those recently described for adaptive/memory NK cells, showing enhanced recall responses following either a viral infection [28] or combined IL-12, IL-15, and IL-18 cytokine stimulations [27]. Although the precise signal transduction mechanisms in adaptive NK cells remain elusive, the enhanced proliferation and cytotoxicity of pre-activated hypoxic NK cells, shown in our study, was dependent on HIF-1α and activation of JAK-STAT pathways [40,41], especially STAT3, downstream of the IL-2R signaling pathway [42]. The importance of STAT3 activation was emphasized by Pawlus and his colleagues in a previous report, where STAT3 knockdown in tumor cells caused inhibition of proliferation, mimicking the phenotype displayed by NK cells after HIF-1α knockdown under hypoxia [35]. Furthermore, STAT3 is also known to be essential in maintaining T cell memory and longevity [43]. Through phosphorylation of STAT3, IL-2 and IL-21 enhanced cytotoxicity both in vivo and in vitro [42,43,44]. Our study also demonstrated STAT3 activation by hypoxia to be essential for the acquisition of survival and functional benefits of NK cells. STAT3 is known to be an activator of human telomerase reverse transcriptase (hTERT) [45], and NK cell senescence from mbIL15-mediated expansion can be restored with hTERT gene modification. Since our data showed a reduction in the expression of p21/p53 tumor suppressors [46], along with a concomitant decrease in p16 mRNAs (data not shown) in hypoxia-exposed NK cells, inhibition of senescence by promoting hTERT activity could also have contributed to the increase in NK cells beyond 20 days of ex-vivo hypoxic NK culture. Therefore, STAT3 could be an important transcription factor in hypoxia that enhances proliferation, longevity, and effector function of human NK cells.

Our mRNA-Seq analysis revealed that hypoxic exposure of NK cells was associated with upregulated HIF-1α target genes and glycolysis-associated genes and downregulated apoptosis- and cell cycle arrest genes; this indicated that they altered their metabolic pathways to adapt to hypoxia. Metabolic reprogramming from oxidative phosphorylation to glycolysis is a common feature of effector lymphocytes, which demand biosynthetic precursors for the proliferation and maintenance of effector molecules [47]. Glycolysis is important for effector functions of activated CD4+ T cells, and limiting aerobic glycolysis drastically reduces the production of IFN-γ and IL-2 [48,49]. Similarly, Finlay and colleagues had reported the mTORC1-HIF-1α pathway to be essential for sustaining glucose metabolism and glycolysis in CD8+ effector T cells [50]. Our current data are in line with these reports considering that proliferating NK cells undergo marked metabolic reprogramming and promote glycolysis under hypoxia. Limiting the rate of glycolysis in NK cells had previously been shown to inhibit IFN-γ production and granzyme B expression [51,52]. Therefore, induction of glycolysis-related genes by HIF-1α may have contributed to the enhanced proliferation and cytotoxicity of pre-activated hypoxic NK cells.

Of note, pre-activated hypoxic NK cells did not significantly alter their activating and/or inhibitory surface-receptors. However, the expression level of *NKp44* activating receptor was found to be elevated both at mRNA and protein levels. The major signal underlying the upregulation of *NKp44* has been shown to be IL-2, which activates PI3K/AKT, Shc, and ERK1/2 through the JAK/STAT pathways [53,54]. Moreover, hypoxia also activates the PI3K/AKT and ERK1/2 pathways and has been shown to be responsible for promoting tumor cell survival [55,56]. Based on these findings, it is plausible that hypoxia amplified the existing IL-2-induced ERK1/2 pathway (Figure 5C), but not the PI3K/AKT pathway (Figure 5D), in proliferating human NK cells, thereby promoting their survival and cytotoxicity via *de-novo* synthesis of NKp44 at the transcriptional level (Figure 7). Our findings are supported by the results from a recent study that activation of cyclin-dependent kinase 1 (CDK1) phosphorylated HIF-1α protein and led to tumor growth [57]. The fact that hypoxia-induced impairment of NK cell cytotoxicity could be restored by IL-2 pre-activation further supports the notion that HIF-1α can synergize with CDK1 and cell cycle-associated target genes in pre-activated proliferating NK cells [11].

Hypoxia is a prominent feature of the tumor microenvironment and constitutes an adverse prognostic factor, especially in solid tumors. While the clinical outcome of adoptive transfer of NK cells shows promising results in the cure of leukemias [58,59], exploitation of NK cells in the therapy of solid tumors is still limited and unsatisfactory [60]. Our current findings highlighted that mature NK cells can resist the detrimental tumor-induced hypoxic immunosuppressive environment if they are in active proliferating state. The proliferative signals of NK cells were sufficient to amplify the hypoxia-induced activation of ERK1/2 and STAT3, thus shifting toward anti-apoptotic and pro-survival pathways. Therefore, for adoptive immunotherapy, NK cells may be conditioned by pre-exposure to hypoxia ex vivo to outperform their tumor killing activity and survival within a hypoxic solid tumor. Our current protocol, involving the pre-activation of NK cells in normoxic culture followed by exposure to hypoxia, could provide a therapeutic solution for successful clinical outcome in patients with advanced stages of solid cancer.

## 4. Materials and Methods

### 4.1. Enrichment and Expansion of NK Cells under Hypoxic Conditions

PBMCs were isolated from 20 mL of healthy donor peripheral blood using Ficoll-Paque PLUS (GE Healthcare, Uppsala, Sweden). 1 × 10^6^ PBMCs were co-cultured with gamma-irradiated (100 Gy) feeder cell lines, KL1 and LCL, at 1:0.5:0.5 ratio in RPMI 1640 medium supplemented with 10% FBS plus recombinant human interleukin-2 (500 U/mL) (rhIL-2, Proleukin; Novartis, Basel, Switzerland). Medium was replaced every 3–4 days with fresh IL-2. At 9 days of co-culture, the percentage of NK cells reach up to 85%. Where indicated, NK cells were purified using MACs isolation kits (Miltenyi, Bergisch Gladbach, North Rhine-Westphalia, Germany) following 9 days of activation with up to 95% purity, prior to exposure of hypoxic condition. The number of PBMCs was assessed by staining the cells with trypan blue at intervals, up to 21 days of culture. For hypoxic treatments, cells were cultured in an anaerobic incubator (<0.5% O_2_, Model 1029 Forma, Thermo Fisher Scientific Inc., Waltham, MA, USA) or in InvivO2 200 Hypoxia workstation (3%, 1.5% O_2_; Baker Ruskinn, Sanford, MN, USA). The absolute number of NK cells was calculated by multiplying the total viable number of cells by the percentage of CD56^+^CD3^−^ cells determined by flow cytometry. Fold expansion was calculated by dividing the number of viable NK cells present at the end of culture by the number of viable NK cells at the beginning of culture. The study was approved by the Institutional Review Board of Korea University Hospital, with donors’ consent (IRB# 1040548-KU-IRB-16-103-A-2).

### 4.2. Cell Culture and Reagents

K562 (human erythroblastoid cell line; ATCC), CEM (human T lymphoblast line; ATCC), A375 (human melanoma cell line; ATCC) and Jurkat, designated as KL-1, (human T lymphoblast line; Korean Cell Line Bank, Seoul, Korea) were cultured in RPMI 1640, supplemented with 10% FBS, 100 U/mL penicillin, and 100 U/mL streptomycin (all from HyClone, Logan, UT, USA) at 37 °C in 5% CO_2_. The EBV-transformed lymphoblastoid B cell line (LCL) was generated from PBMCs, using EBV supernatant from B95-8 cells. JSI-124 (Calbiochem, San Diego, CA, USA) were dissolved in dimethyl sulfoxide (DMSO) and stored at −80 °C.

### 4.3. Flow Cytometry

Anti-human CD3 (OKT3), CD56 (MEM188), CD11a (HI111), ICAM-1 (HA58), NKG2D (5C6), CD2 (RPA-2.10), CD25 (BC96), Perforin (dG9), Granzyme B (GB11), CD16 (CB16), CD69 (FN50), and FAS (15A7) mAbs were purchased from eBioscience (San Diego, CA, USA). Anti-human 2B4 (clone 2–69), NKp44 (P44-8.1), CD122 (Mik-β3), CD132 (AG184), NKp30 (P30-15), NKp46 (9E2), DNAM-1 (DX11), KIR-NKAT2 (DX27), CD94 (HP-3D9), NKB1 (DX9), CD158a (HP-3E4), and CD158b (CH-L) mAbs were purchased from BD Pharmingen (San Jose, CA, USA). PE-conjugated anti-human HIF-1α (546-16) were purchased from BioLegend (San Diego, CA, USA). For surface staining, cells were stained with the indicated PE-, FITC-, APC-, or PerCP-conjugated mAbs in 100 μL of FACS buffer (BD Biosciences, Franklin Lakes, NJ, USA). To detect NKp44L, target cells were incubated with 20 μg/mL of NKp44-Ig fusion proteins (R&D Systems, Minneapolis, MN, USA). Cells were then incubated with Fcγ-fragment-specific conjugated affinity-purified F(ab′)_2_ fragments of goat anti-human IgG (1/50; Jackson ImmunoResearch, West Grove, PA, USA). Flow cytometry was performed with a FACSCantoΙΙ (BD Biosciences) and data were analyzed with FlowJo software (Tree Star, Ashland, OR, USA).

### 4.4. Proliferation and Apoptosis Assay

To determine NK cell proliferation, cells cultured under normoxic and hypoxic conditions were harvested and incubated for 20 min with CD3-PercP and CD56-APC. Intranuclear fixation was performed following the manufacturer’s instruction (Foxp3/Transcription Factor Fixation/Permeabilization, eBioscience). After washing and resuspending the cells with FACS buffer, they were stained with Ki67-PE for 20 min in the dark at room temperature. Cells were washed with FACS buffer and the rate of proliferation was detected in the cells stained positive for Ki67 (SolA15, eBioscience) and CD56 with FACSCantoII and analyses were performed using the FlowJo software (Tree Star, Ashland, OR, USA). Apoptotic cells were detected by double staining with annexin V-APC/7-Amino- Actinomycin D (7AAD, BD Pharmingen) according to the manufacturer’s instruction. Briefly, after 18-d culture, under normoxic and hypoxic conditions, cells were washed with PBS and stained with CD3-FITC and CD56-PE for 20 min at 4 °C. After washing the cells with FACS buffer and resuspending in 100 μL annexin V-binding buffer (BD Pharmingen), they were stained with 2 μL annexin V-APC and 7AAD for 15 min in the dark at room temperature. An additional 300 μL annexin V-binding buffer was added, and 7AAD-positive and annexin V-positive cells were recorded on FACSCantoII and analyses were performed using the FlowJo software (Tree Star).

### 4.5. 10-N Nonyl-Acridine Orange (NAO) Staining for Measuring Mitochondrial Mass

Mitochondria mass was measured according to the manufacturer’s instruction. Briefly, after 18-d culture, under normoxic and hypoxic conditions, cells were washed with PBS and stained with CD3-FITC, CD56-PE, and 50 nM NAO (Thermo Fisher Scientific) for 30 min at 37 °C. After washing and resuspending with FACS buffer, the cells were analyzed by flow cytometry on a BD FACSCantoII.

### 4.6. Cr Release Assay

^51^Cr release assay was performed, as described previously [61,62]. In brief, K562, A375, and CEM target cells were labeled with ^51^Cr (Perkin Elmer, Boston, MA, USA) at 50 μCi/5 × 10^5^ cells and incubated at 5 × 10^3^ cells/well with serially diluted NK cells for 4 h. In some experiments, the assay was performed in presence of 5 μg/mL of blocking anti-NKp44 mAbs or isotype controls. The supernatant was assayed using a γ-scintillation counter (Perkin Elmer). Percent specific lysis was calculated as follows: 100 × (experimental release–spontaneous release)/(maximum release–spontaneous release).

### 4.7. Cytotoxicity Assay

Tumor killing activity of purified NK cells was measured using LDH (lactate dehydrogenase) cytotoxicity detection kit (Roche Diagnostics). The percentage of specific lysis was calculated as follows: [((abs 492 nm effector/target mix − abs 492 nm effector only) − abs 492 nm spontaneous)/(abs 492 nm maximum − abs 492 nm spontaneous)] × 100.

### 4.8. CD107a Granule Release Assay and Intracellular Cytokine Staining

The CD107a assay was performed following a previously described procedure [30]. Briefly, resting or expanded NK cells under 20%, 1.5%, and 0.5% pO_2_ conditions were co-cultured with K562, A375, and CEM cells with E:T ratio of 1:1 in a round-bottomed 96-well plate. After 1-h incubation, anti-CD107a mAb conjugated with FITC was treated at 2.5 μL/well, and Golgistop (BD Pharmingen) was added to the culture in the last 5 h. The samples were harvested and incubated with CD3-PercP and CD56-APC for 20 min, after which they were fixed and permeabilized using Cytofix/Cytoperm intracellular staining kits (BD Pharmingen). Intracellular staining of NK cells was performed with anti-IFN-γ-PE mAb for an additional 20 min. Flow cytometry was performed with FACSCantoII (BD Bioscience) and the data were analyzed with the FlowJo (Tree Star) software.

### 4.9. Transduction of NK Cells with Lentiviral Vectors Encoding shHIF-1α

shCtrl and shHIF-1α NK cells were generated by infecting NK cells with lentivirus encoding shRNA against human HIF-1α (5′-CCAGTTATGATTGTGAAGTTA-3′) and control (5′-CCTAAGGTTAAGTCGCCCTCG-3′) using the pLKO.1 lentiviral vector system (Addgene, Cambridge, MA, USA). Knockdown was confirmed using flow cytometry of intracellular HIF-1α. Briefly, lentivirus transduced NK cells were stained with CD3-PercP and CD56-APC for 20 min. Cells were fixed and permeabilized using Cytofix/Cytoperm intracellular staining kits (BD Pharmingen), followed by staining with anti-HIF-1α-PE mAb (546-16, BioLegend) for an additional 20 min. Flow cytometry was performed with FACSCantoII (BD Bioscience) and the data were analyzed with FlowJo (Tree Star) software.

### 4.10. In-Vivo Cytotoxicity Assay

Seven to 8-week-old female NSG (NOD/SCID/IL-2Rγ-/-) mice (Jackson Laboratory, Bar Harbor, ME) were maintained in Korea University (Seoul, Korea) animal housing facility under specific pathogen-free conditions. All animal experiments were approved by Institutional Animal Care and Use Committee, Korea University, and were performed in accordance with national and institutional guidelines (IACUC# KOREA-2017-0066-C1). To determine the in-vivo cytotoxicity of expanded NK cells, CEM cells were labeled with PKH26 as per manufacturer’s instructions (Sigma, St. Louis, MO, USA). Five million CEM cells in 250 μL PBS were injected intraperitoneally (i.p.). After 30 min, 1 × 10^7^ expanded human NK cells in 800 μL PBS, under normoxia and hypoxia, were injected i.p. After 2 days, the mice were sacrificed and peritoneal cells were recovered. Tumor cells were distinguished by PKH26 labeling. Data were acquired on FACSCantoII and analyses were performed using the FlowJo (Tree Star) software.

### 4.11. Western Blot Analysis

For preparation of whole cell extracts, cells were harvested, washed with PBS, and lysed with RIPA buffer containing 50 mM Tris pH 7.4, 150 mM NaCl, 1% NP-40, 0.5% deoxycholate, 1 mM phenyl methylsulfonyl fluoride (PMSF), 1 µg/mL leupeptin, 1 µg/mL aprotinin, 25 mM β-glycerophosphate, 25 mM NaF, 10 mM EGTA, 1 mM DTT, 1 mM Na_3_VO_4_, and 0.1% sodium dodecyl sulfate (SDS) for 20 min in ice. The lysates were centrifuged at 13,000× *g* for 10 min at 4 °C. Protein concentration of the supernatant was determined by Bradford assay (Bio-Rad, Hercules, CA, USA). Equal amount of each protein lysate was separated by 7–15% SDS-PAGE and transferred to polyvinylidene fluoride (PVDF) membrane in semi-dry state using Trans-Blot SD (Bio-Rad, Hercules, CA, USA). The membrane was blocked with 5% skim milk in Tris-buffered saline containing Tween-20 for 2 h at room temperature. The membrane was incubated with primary antibodies at 4 °C overnight, followed by incubation with horseradish peroxidase (HRP)-conjugated secondary antibodies. Proteins were visualized by an enhanced chemiluminescent (ECL) substrate kit (GE Healthcare, Pittsburgh, PA, USA). Antibodies used in the study were anti-human HIF-1α (BD Biosciences), anti-β-actin (Sigma), anti-α tubulin (Cell Signaling Technology, Danvers, MA, USA), anti-p21, anti-p53 (Santa Cruz Biotechnology, Dallas, TX, USA), anti-Bax, anti-Bim, anti-cIAP1, anti-cIAP2, anti-XIAP, anti-phospho STAT3, anti-STAT3, anti-phospho-p44/p42 MAPK (Erk1/2) (Thr202/Tyr204), anti-p44/p42 MAPK (Erk1/2), anti-phopho Akt (Ser473), and anti-Akt (Cell Signaling Technology). The secondary antibodies included anti-rabbit or anti-mouse IgG obtained from Santa Cruz Biotechnology.

### 4.12. Real-Time Quantitative Reverse Transcription-Polymerase Chain Reaction (qRT-PCR)

Total RNA was isolated using TRIzol (Molecular Research Center, Cincinnati, OH, USA). RNA (1 µg) was reverse transcribed, using M-MLV reverse transcriptase (Promega, Madison, WI, USA), dNTPs, and random primers. One microgram of isolated total RNA was used for reverse transcription. qRT-PCR was performed using the 7000 sequence detection system (Applied Biosystems, Warrington, UK) [63,64]. The expression level of 18S rRNA was used for normalization.

### 4.13. mRNA-Sequencing and Data Analysis

Poly(A) mRNA isolation from total RNA of purified NK cells and subsequent fragmentation were performed using the Illumina TruSeq RNA Sample Prep Kit v2. The adaptor-ligated libraries were sequenced using an Illumina HiSeq 2500 (Macrogen, Seoul, Korea). From the resulting read sequences for each sample, adapter sequences (TruSeq universal and indexed adapters) were removed using Cutadapt software (version 1.10) [65]. The remaining reads were then aligned to the *Homo sapiens* reference genome (GRCh38) using TopHat2 software (version 2.1.1) with default parameters [66]. After the alignment, we counted the number of reads mapped to the gene features (GTF file of GRCm38.91) using HTSeq. Read counts for the samples in each condition were then normalized using trimmed mean of M-values (TMM) normalization in the edgeR package [67].

### 4.14. Identification of DEGs

To identify DEGs, we performed a statistical hypothesis test, as described previously [68]. To identify the cellular processes represented by the DEGs, we performed enrichment analysis of GOBPs using DAVID software [69] and selected the GOBPs with *p*-value < 0.05 as the processes enriched by the DEGs.

### 4.15. Statistics

Statistical analyses were performed using either Student’s *t*-test for comparison between two samples, or two-way ANOVA followed by Sidak’s test for multiple comparisons, using GraphPad Prism 5.0. (GraphPad Software, Northampton, MA, USA). *, *p* < 0.05; **, *p* < 0.01; ***, *p* < 0.001. Error bars represent SEM or standard deviation

## 5. Conclusions

Hypoxic exposure on pre-activated NK cells triggered HIF-1α-dependent pathways to initiate coordinated regulation of cell cycle, apoptosis, and cytotoxicity. Our results uncover a previously unidentified role of HIF-1α-mediated metabolic reprogramming that can reverse impaired NK effector phenotypes to generate requisite numbers of functionally robust NK cells for adoptive cellular therapy.

## Data Availability

The data sets generated during the current study are available from the corresponding authors on reasonable requests.

## Supplementary Materials

The following are available online at https://www.mdpi.com/article/10.3390/cancers13081904/s1, Figure S1A and S1B. Replicates of NK cell expansion under 20%, 3%, 1.5%, and 0.5% pO_2_ conditions. Figure S1C. Replicates of NK cell expansion under 20% and 1.5% (3) pO_2_ conditions. Figure S1D. Replicates of NK cell expansion under 20% and 1.5% (5) pO_2_ conditions. Figure S1E. Replicates of NK cell expansion under 20% and 1.5% (7) pO_2_ conditions. Figure S1F. Replicates of NK cell expansion under 20% and 1.5% (9) pO_2_ conditions. Figure S1G. Replicates of NK cell expansion under 20% and 1.5% (13) pO_2_ conditions. Figure S1H. Replicates of NK cell expansion under 20% and 1.5% (17) pO_2_ conditions. Figure S2. Highly purified NK cells from day 9 of culture showed enhanced proliferation in hypoxia-switched culture condition. Figure S3. HIF-2α protein level was not significantly changed in the pre-activated hypoxic NK cells. Figure S4. Original whole blots of western blot figures. Figure S5. NKp44 is the only activating receptor upregulated at the level of mRNA in pre-activated hypoxic NK cells. Table S1. 496 DEGs. For each DEG, entrez ID, gene symbol and description, log2-fold-change of mRNA expression levels between normoxic and pre-activated hypoxic NK cells are shown.

## References

[1] Guillerey C., Huntington N.D., Smyth M.J. (2016). Targeting natural killer cells in cancer immunotherapy. Nat. Immunol..

[2] Myers J.A., Miller J.S. (2020). Exploring the NK cell platform for cancer immunotherapy. Nat. Rev. Clin. Oncol..

[3] Morvan M.G., Lanier L.L. (2016). NK cells and cancer: You can teach innate cells new tricks. Nat. Rev. Cancer.

[4] Paul S., Kulkarni N., Shilpi, Lal G. (2016). Intratumoral natural killer cells show reduced effector and cytolytic properties and control the differentiation of effector Th1 cells. OncoImmunology.

[5] Paul S., Lal G. (2017). The Molecular Mechanism of Natural Killer Cells Function and Its Importance in Cancer Immunotherapy. Front. Immunol..

[6] Barrow A.D., Edeling M.A., Trifonov V., Luo J., Goyal P., Bohl B., Bando J.K., Kim A.H., Walker J., Andahazy M. (2018). Natural Killer Cells Control Tumor Growth by Sensing a Growth Factor. Cell.

[7] Hasmim M., Messai Y., Ziani L., Thiery J., Bouhris J.H., Noman M.Z., Chouaib S. (2015). Critical Role of Tumor Microenvironment in Shaping NK Cell Functions: Implication of Hypoxic Stress. Front Immunol.

[8] Melaiu O., Lucarini V., Cifaldi L., Fruci D. (2019). Influence of the Tumor Microenvironment on NK Cell Function in Solid Tumors. Front. Immunol..

[9] Shin M.H., Kim J., Lim S.A., Kim J., Kim S.-J., Lee K.-M. (2020). NK Cell-Based Immunotherapies in Cancer. Immune Netw..

[10] Balsamo M., Manzini C., Pietra G., Raggi F., Blengio F., Mingari M.C., Varesio L., Moretta L., Bosco M.C., Vitale M. (2013). Hypoxia downregulates the expression of activating receptors involved in NK-cell-mediated target cell killing without affecting ADCC. Eur. J. Immunol..

[11] Sarkar S., Germeraad W.T., Rouschop K.M., Steeghs E.M., van Gelder M., Bos G.M., Wieten L. (2013). Hypoxia induced impairment of NK cell cytotoxicity against multiple myeloma can be overcome by IL-2 activation of the NK cells. PLoS ONE.

[12] Solocinski K., Padget M.R., Fabian K.P., Wolfson B., Cecchi F., Hembrough T., Benz S.C., Rabizadeh S., Soon-Shiong P., Schlom J. (2020). Overcoming hypoxia-induced functional suppression of NK cells. J. Immunother. Cancer.

[13] Nakayama K., Kataoka N. (2019). Regulation of Gene Expression under Hypoxic Conditions. Int. J. Mol. Sci..

[14] Parodi M., Raggi F., Cangelosi D., Manzini C., Balsamo M., Blengio F., Eva A., Varesio L., Pietra G., Moretta L. (2018). Hypoxia Modifies the Transcriptome of Human NK Cells, Modulates Their Immunoregulatory Profile, and Influences NK Cell Subset Migration. Front. Immunol..

[15] Choudhry H., Harris A.L. (2018). Advances in Hypoxia-Inducible Factor Biology. Cell Metab..

[16] Baba Y., Nosho K., Shima K., Irahara N., Chan A.T., Meyerhardt J.A., Chung D.C., Giovannucci E.L., Fuchs C.S., Ogino S. (2010). HIF1A overexpression is associated with poor prognosis in a cohort of 731 colorectal cancers. Am. J. Pathol..

[17] Jögi A., Ehinger A., Hartman L., Alkner S. (2019). Expression of HIF-1α is related to a poor prognosis and tamoxifen resistance in contralateral breast cancer. PLoS ONE.

[18] Birner P., Schindl M., Obermair A., Plank C., Breitenecker G., Oberhuber G. (2000). Overexpression of hypoxia-inducible factor 1alpha is a marker for an unfavorable prognosis in early-stage invasive cervical cancer. Cancer Res..

[19] Liu Q., Cao P. (2015). Clinical and prognostic significance of HIF-1α in glioma patients: A meta-analysis. Int. J. Clin. Exp. Med..

[20] Zhao X., Gao S., Ren H., Sun W., Zhang H., Sun J., Yang S., Hao J. (2014). Hypoxia-inducible factor-1 promotes pancreatic ductal adenocarcinoma invasion and metastasis by activating transcription of the actin-bundling protein fascin. Cancer Res..

[21] Naik P.P., Birbrair A., Bhutia S.K. (2019). Mitophagy-driven metabolic switch reprograms stem cell fate. Cell. Mol. Life Sci..

[22] O’Sullivan T.E., Johnson L.R., Kang H.H., Sun J.C. (2015). BNIP3- and BNIP3L-Mediated Mitophagy Promotes the Generation of Natural Killer Cell Memory. Immunity.

[23] Nakhle J., Rodriguez A.M., Vignais M.L. (2020). Multifaceted Roles of Mitochondrial Components and Metabolites in Metabolic Diseases and Cancer. Int. J. Mol. Sci..

[24] Macleod K.F. (2020). Mitophagy and Mitochondrial Dysfunction in Cancer. Annu. Rev. Cancer Biol..

[25] Zhang H., Li H., Xi H.S., Li S. (2012). HIF1alpha is required for survival maintenance of chronic myeloid leukemia stem cells. Blood.

[26] Krzywinska E., Kantari-Mimoun C., Kerdiles Y., Sobecki M., Isagawa T., Gotthardt D., Castells M., Haubold J., Millien C., Viel T. (2017). Loss of HIF-1α in natural killer cells inhibits tumour growth by stimulating non-productive angiogenesis. Nat. Commun..

[27] Romee R., Rosario M., Berrien-Elliott M.M., Wagner J.A., Jewell B.A., Schappe T., Leong J.W., Abdel-Latif S., Schneider S.E., Willey S. (2016). Cytokine-induced memory-like natural killer cells exhibit enhanced responses against myeloid leukemia. Sci. Transl. Med..

[28] Sun J.C., Beilke J.N., Lanier L.L. (2009). Adaptive immune features of natural killer cells. Nature.

[29] Brillantes M., Beaulieu A.M. (2020). Memory and Memory-Like NK Cell Responses to Microbial Pathogens. Front. Cell. Infect. Microbiol..

[30] Lim S.A., Kim T.J., Lee J.E., Sonn C.H., Kim K., Kim J., Choi J.G., Choi I.K., Yun C.O., Kim J.H. (2013). Ex vivo expansion of highly cytotoxic human NK cells by cocultivation with irradiated tumor cells for adoptive immunotherapy. Cancer Res..

[31] Semenza G.L. (2014). Oxygen sensing, hypoxia-inducible factors, and disease pathophysiology. Annu. Rev. Pathol..

[32] Nagao A., Kobayashi M., Koyasu S., Chow C.C.T., Harada H. (2019). HIF-1-Dependent Reprogramming of Glucose Metabolic Pathway of Cancer Cells and Its Therapeutic Significance. Int. J. Mol. Sci..

[33] Richard D.E., Berra E., Gothie E., Roux D., Pouyssegur J. (1999). p42/p44 mitogen-activated protein kinases phosphorylate hypoxia-inducible factor 1alpha (HIF-1alpha) and enhance the transcriptional activity of HIF-1. J. Biol. Chem..

[34] Kidger A.M., Rushworth L.K., Stellzig J., Davidson J., Bryant C.J., Bayley C., Caddye E., Rogers T., Keyse S.M., Caunt C.J. (2017). Dual-specificity phosphatase 5 controls the localized inhibition, propagation, and transforming potential of ERK signaling. Proc. Natl. Acad. Sci. USA.

[35] Pawlus M.R., Wang L., Hu C.J. (2014). STAT3 and HIF1alpha cooperatively activate HIF1 target genes in MDA-MB-231 and RCC4 cells. Oncogene.

[36] Wang X., Lee D.A., Wang Y., Wang L., Yao Y., Lin Z., Cheng J., Zhu S. (2013). Membrane-bound interleukin-21 and CD137 ligand induce functional human natural killer cells from peripheral blood mononuclear cells through STAT-3 activation. Clin. Exp. Immunol..

[37] Garg S.M., Vakili M.R., Molavi O., Lavasanifar A. (2017). Self-Associating Poly(ethylene oxide)-block-poly(α-carboxyl-ε-caprolactone) Drug Conjugates for the Delivery of STAT3 Inhibitor JSI-124: Potential Application in Cancer Immunotherapy. Mol. Pharm..

[38] Lee K.M., Forman J.P., McNerney M.E., Stepp S., Kuppireddi S., Guzior D., Latchman Y.E., Sayegh M.H., Yagita H., Park C.K. (2006). Requirement of homotypic NK-cell interactions through 2B4(CD244)/CD48 in the generation of NK effector functions. Blood.

[39] Siewiera J., Gouilly J., Hocine H.R., Cartron G., Levy C., Al-Daccak R., Jabrane-Ferrat N. (2015). Natural cytotoxicity receptor splice variants orchestrate the distinct functions of human natural killer cell subtypes. Nat. Commun..

[40] Wang G., Qian P., Jackson F.R., Qian G., Wu G. (2008). Sequential activation of JAKs, STATs and xanthine dehydrogenase/oxidase by hypoxia in lung microvascular endothelial cells. Int. J. Biochem. Cell Biol..

[41] Banerjee K., Resat H. (2016). Constitutive activation of STAT3 in breast cancer cells: A review. Int. J. Cancer.

[42] Ross S.H., Cantrell D.A. (2018). Signaling and Function of Interleukin-2 in T Lymphocytes. Annu. Rev. Immunol..

[43] Deenick E.K., Pelham S.J., Kane A., Ma C.S. (2018). Signal Transducer and Activator of Transcription 3 Control of Human T and B Cell Responses. Front. Immunol..

[44] Bhatt S., Matthews J., Parvin S., Sarosiek K.A., Zhao D., Jiang X., Isik E., Letai A., Lossos I.S. (2015). Direct and immune-mediated cytotoxicity of interleukin-21 contributes to antitumor effects in mantle cell lymphoma. Blood.

[45] Dratwa M., Wysoczańska B., Łacina P., Kubik T., Bogunia-Kubik K. (2020). TERT—Regulation and Roles in Cancer Formation. Front. Immunol..

[46] Georgakilas A.G., Martin O.A., Bonner W.M. (2017). p21: A Two-Faced Genome Guardian. Trends Mol. Med..

[47] Donnelly R.P., Finlay D.K. (2015). Glucose, glycolysis and lymphocyte responses. Mol. Immunol..

[48] Chang C.H., Curtis J.D., Maggi L.B., Faubert B., Villarino A.V., O’Sullivan D., Huang S.C., van der Windt G.J., Blagih J., Qiu J. (2013). Posttranscriptional control of T cell effector function by aerobic glycolysis. Cell.

[49] Salmond R.J. (2018). mTOR Regulation of Glycolytic Metabolism in T Cells. Front. Cell Dev. Biol..

[50] Finlay D.K., Rosenzweig E., Sinclair L.V., Feijoo-Carnero C., Hukelmann J.L., Rolf J., Panteleyev A.A., Okkenhaug K., Cantrell D.A. (2012). PDK1 regulation of mTOR and hypoxia-inducible factor 1 integrate metabolism and migration of CD8+ T cells. J. Exp. Med..

[51] Donnelly R.P., Loftus R.M., Keating S.E., Liou K.T., Biron C.A., Gardiner C.M., Finlay D.K. (2014). mTORC1-dependent metabolic reprogramming is a prerequisite for NK cell effector function. J. Immunol..

[52] Wang Z., Guan D., Wang S., Chai L.Y.A., Xu S., Lam K.P. (2020). Glycolysis and Oxidative Phosphorylation Play Critical Roles in Natural Killer Cell Receptor-Mediated Natural Killer Cell Functions. Front. Immunol..

[53] Pol J.G., Caudana P., Paillet J., Piaggio E., Kroemer G. (2020). Effects of interleukin-2 in immunostimulation and immunosuppression. J. Exp. Med..

[54] Pazina T., Shemesh A., Brusilovsky M., Porgador A., Campbell K.S. (2017). Regulation of the Functions of Natural Cytotoxicity Receptors by Interactions with Diverse Ligands and Alterations in Splice Variant Expression. Front. Immunol..

[55] Alvarez-Tejado M., Naranjo-Suarez S., Jimenez C., Carrera A.C., Landazuri M.O., del Peso L. (2001). Hypoxia induces the activation of the phosphatidylinositol 3-kinase/Akt cell survival pathway in PC12 cells: Protective role in apoptosis. J. Biol. Chem..

[56] Hoxhaj G., Manning B.D. (2020). The PI3K–AKT network at the interface of oncogenic signalling and cancer metabolism. Nat. Rev. Cancer.

[57] Warfel N.A., Dolloff N.G., Dicker D.T., Malysz J., El-Deiry W.S. (2013). CDK1 stabilizes HIF-1alpha via direct phosphorylation of Ser668 to promote tumor growth. Cell Cycle.

[58] Berrien-Elliott M.M., Romee R., Fehniger T.A. (2015). Improving natural killer cell cancer immunotherapy. Curr. Opin. Organ. Transpl..

[59] Hodgins J.J., Khan S.T., Park M.M., Auer R.C., Ardolino M. (2019). Killers 2.0: NK cell therapies at the forefront of cancer control. J. Clin. Investig..

[60] Pietra G., Vitale C., Pende D., Bertaina A., Moretta F., Falco M., Vacca P., Montaldo E., Cantoni C., Mingari M.C. (2016). Human natural killer cells: News in the therapy of solid tumors and high-risk leukemias. Cancer Immunol. Immunother..

[61] Kim T.J., Kim N., Kang H.J., Kim E.O., Kim S.T., Ahn H.S., Bluestone J.A., Lee K.M. (2010). FK506 causes cellular and functional defects in human natural killer cells. J. Leukoc. Biol..

[62] Lim S.A., Kim J., Jeon S., Shin M.H., Kwon J., Kim T.-J., Im K., Han Y., Kwon W., Kim S.-W. (2019). Defective Localization With Impaired Tumor Cytotoxicity Contributes to the Immune Escape of NK Cells in Pancreatic Cancer Patients. Front. Immunol..

[63] Lee H.Y., Yang E.G., Park H. (2013). Hypoxia enhances the expression of prostate-specific antigen by modifying the quantity and catalytic activity of Jumonji C domain-containing histone demethylases. Carcinogenesis.

[64] Moon Y., Lee S., Park B., Park H. (2018). Distinct hypoxic regulation of preadipocyte factor-1 (Pref-1) in preadipocytes and mature adipocytes. Biochim. Biophys. Acta.

[65] Martin M. (2011). Cutadapt removes adapter sequences from high-throughput sequencing reads. Embnet. J..

[66] Trapnell C., Pachter L., Salzberg S.L. (2009). TopHat: Discovering splice junctions with RNA-Seq. Bioinformatics.

[67] Robinson M.D., Oshlack A. (2010). A scaling normalization method for differential expression analysis of RNA-seq data. Genome Biol..

[68] Chae S., Ahn B.Y., Byun K., Cho Y.M., Yu M.H., Lee B., Hwang D., Park K.S. (2013). A systems approach for decoding mitochondrial retrograde signaling pathways. Sci. Signal..

[69] Huang da W., Sherman B.T., Lempicki R.A. (2009). Systematic and integrative analysis of large gene lists using DAVID bioinformatics resources. Nat. Protoc..

