# Supplementary material for: Hypoxia-Driven HIF-1α Activation Reprograms Pre-Activated NK Cells towards Highly Potent Effector Phenotypes via ERK/STAT3 Pathways
Source: Cancers (Basel). 2021 Apr 15;13(8):1904. doi: 10.3390/cancers13081904 (PMC8071270; doi:10.3390/cancers13081904)
Supplement: Supplementary file 1 [file cancers-13-01904-s001.zip › cancers-1170529-supplementary-final 20210421_optimize.pdf]

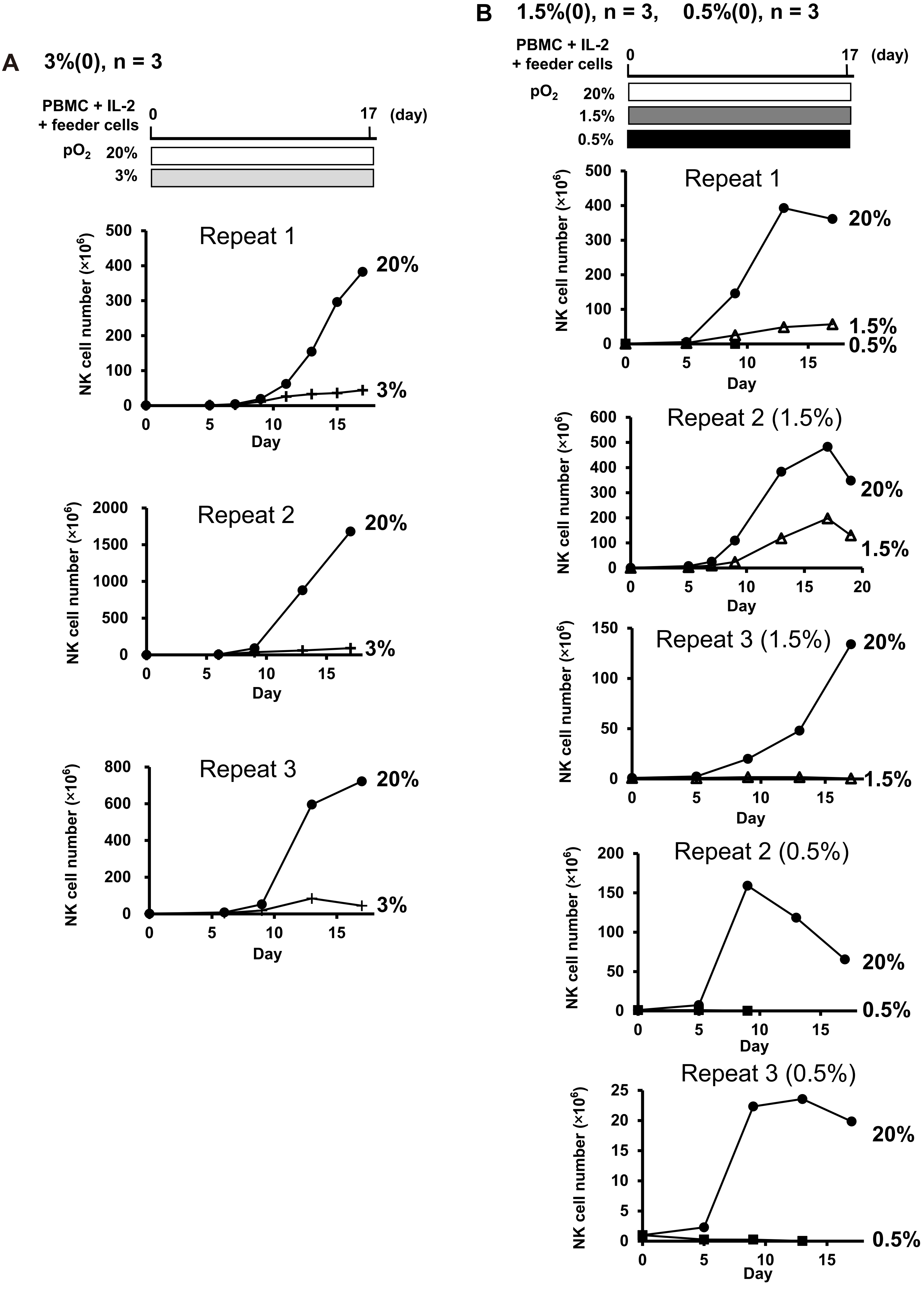

Figure S1A and S1B. Replicates of NK cell expansion under 20%, 3%, 1.5 %, and 0.5% pO_2 conditions.

C **1.5%(3), n = 3**

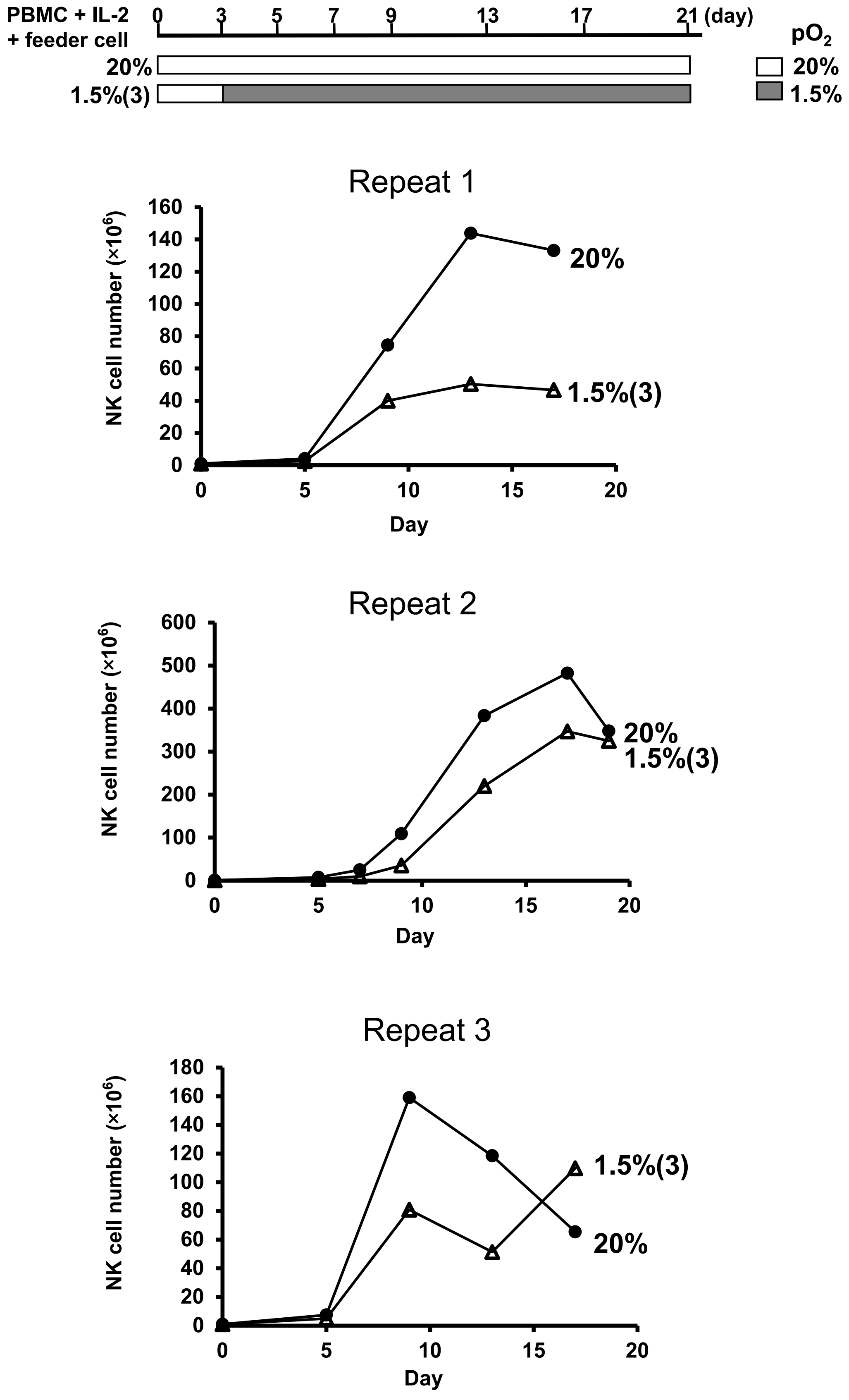

Figure S1C. Replicates of NK cell expansion under 20% and 1.5%(3) pO_2 conditions.

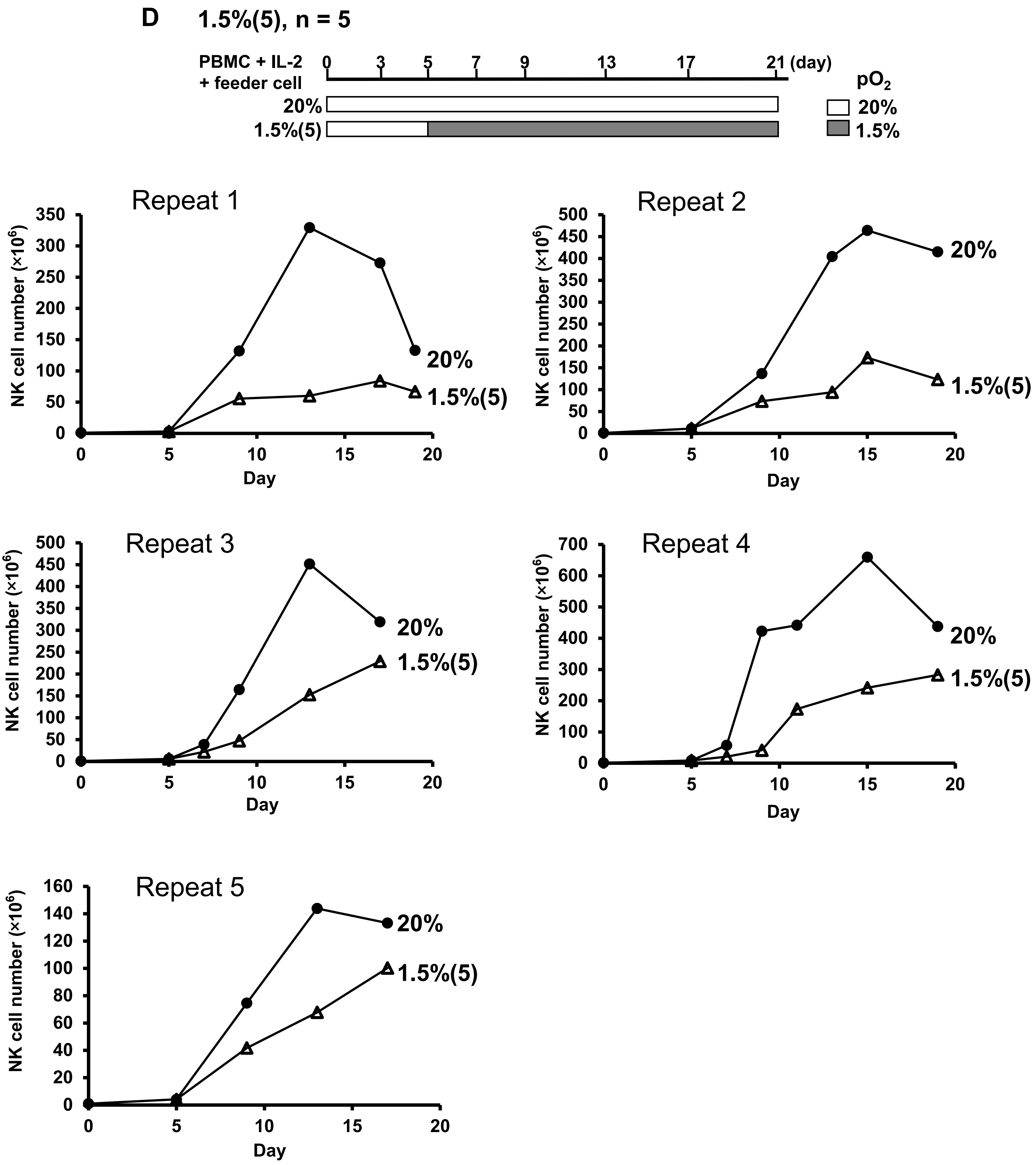

Figure S1D. Replicates of NK cell expansion under 20% and 1.5%(5) pO_2 conditions.

E 1.5%(7), n = 3

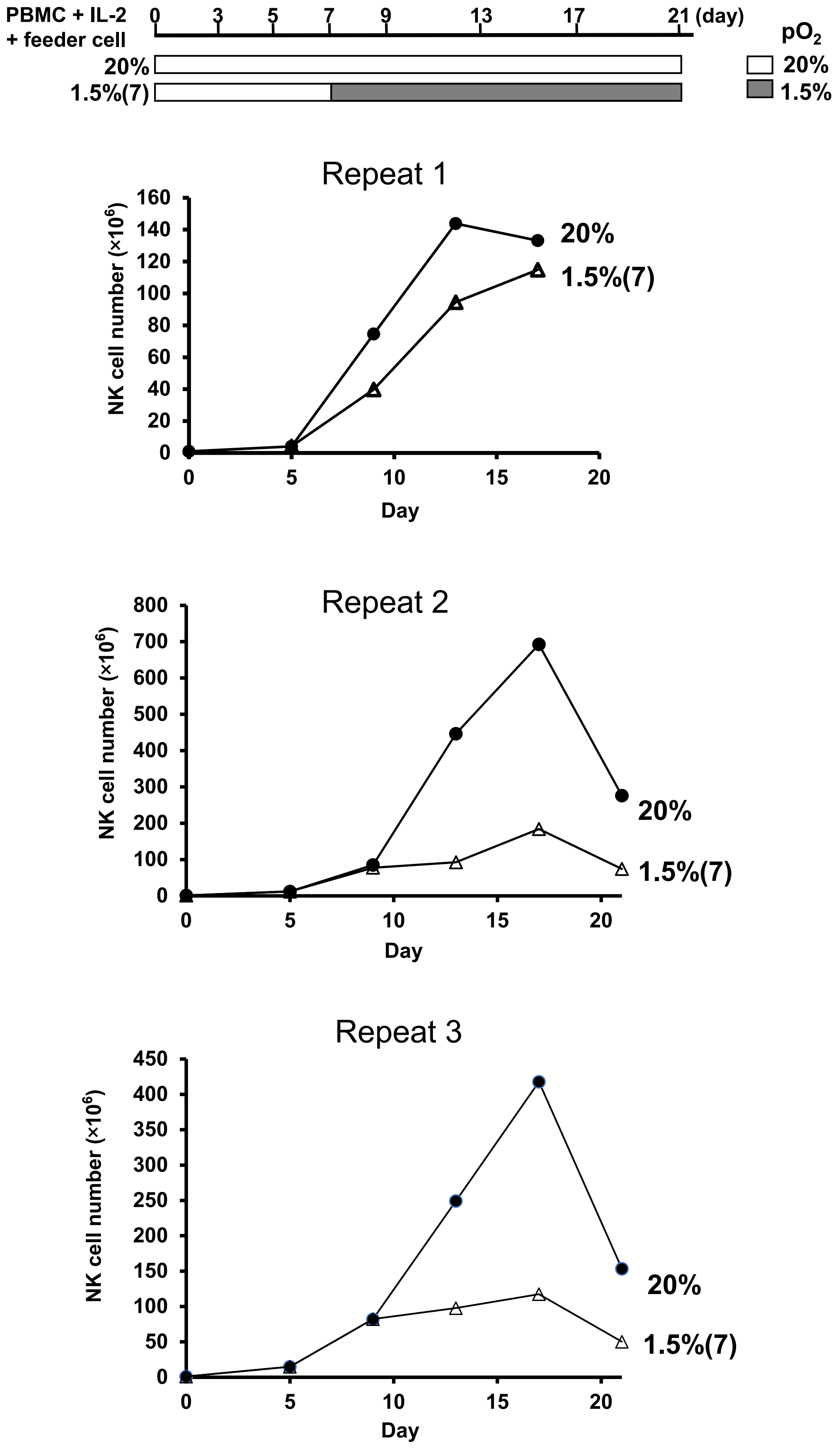

Figure S1E. Replicates of NK cell expansion under 20% and 1.5%(7) pO_2 conditions.

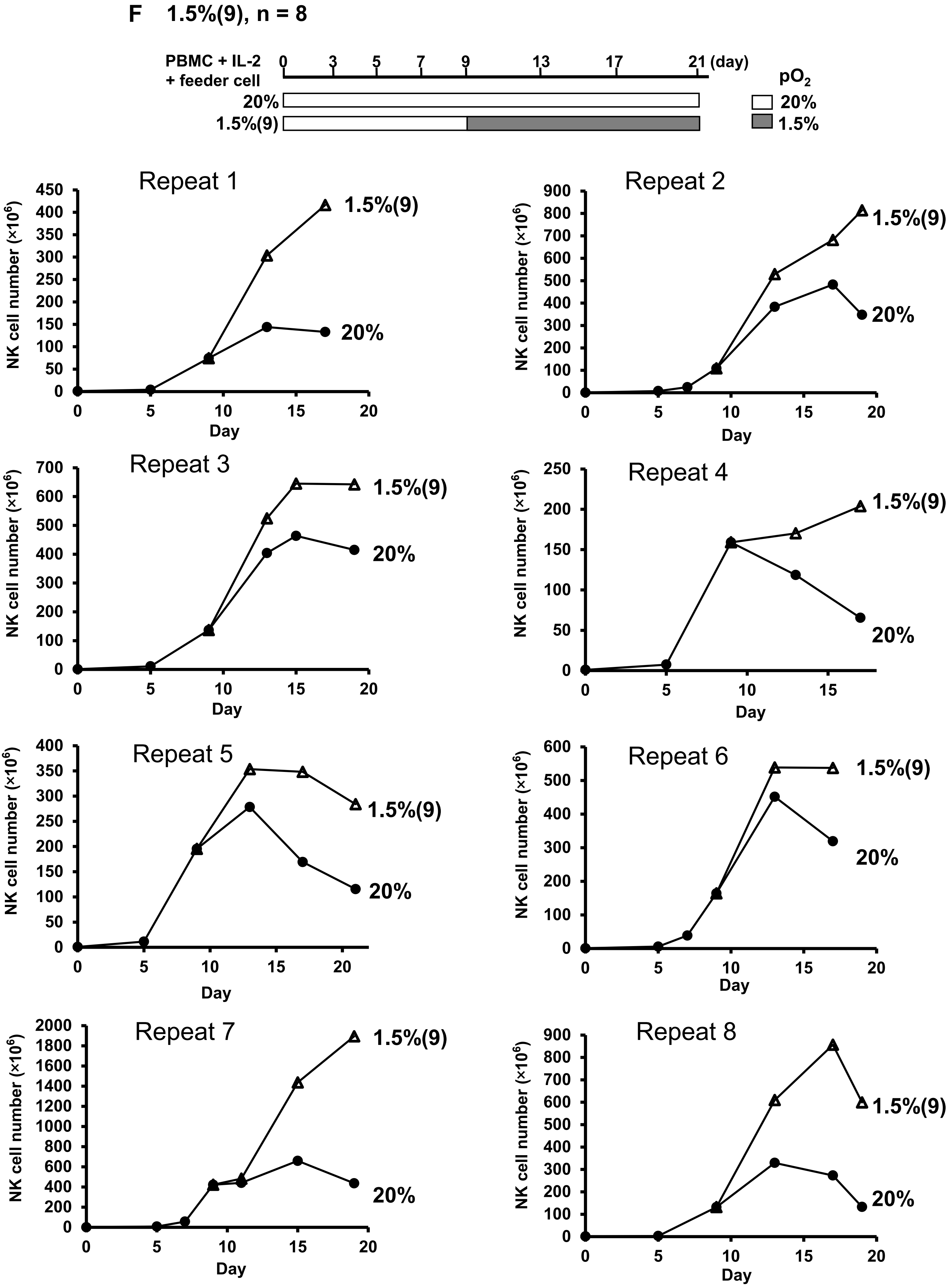

Figure S1F. Replicates of NK cell expansion under 20% and 1.5%(9) pO_2 conditions.

G 1.5%(13), n = 3

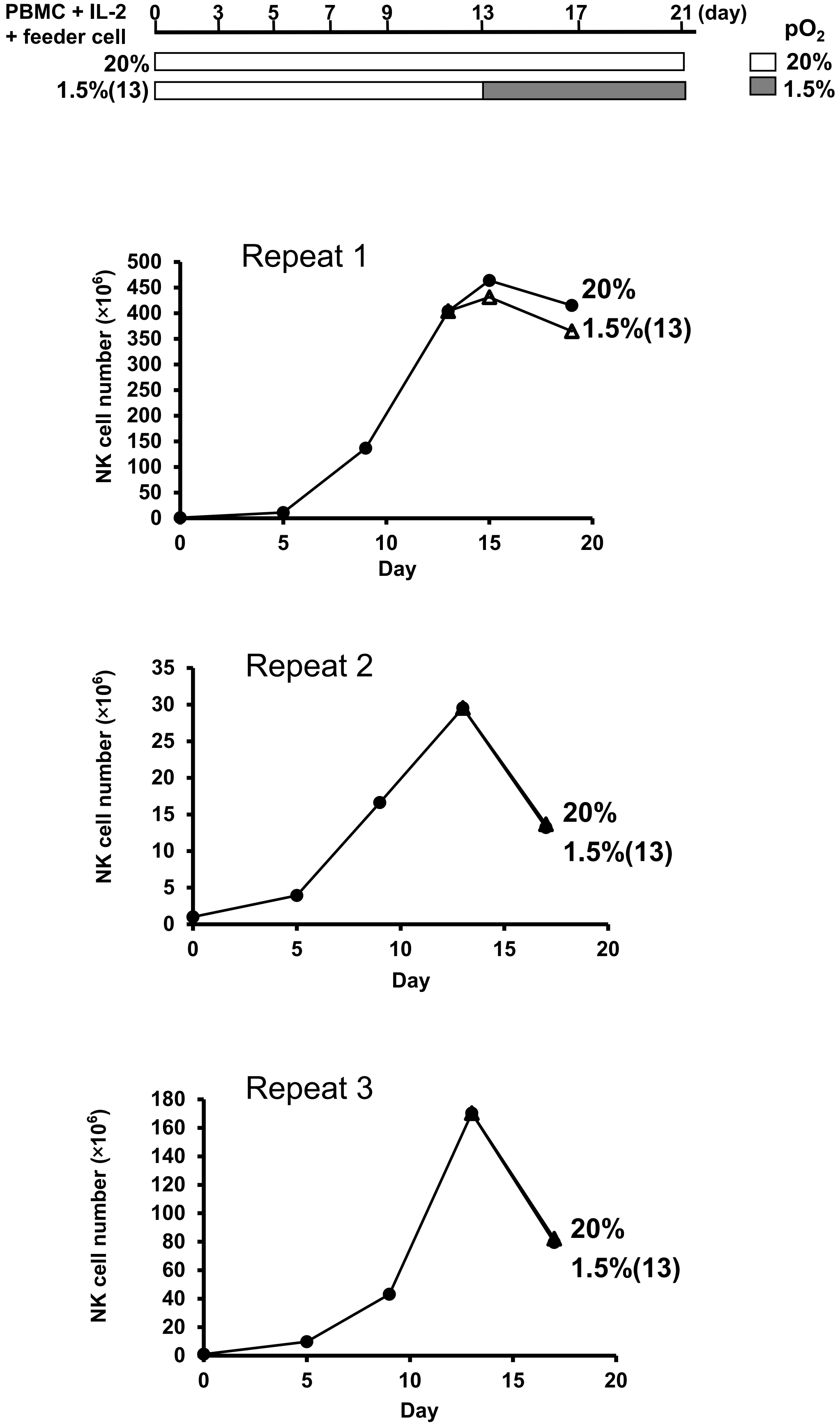

Figure S1G. Replicates of NK cell expansion under 20% and 1.5%(13) pO_2 conditions.

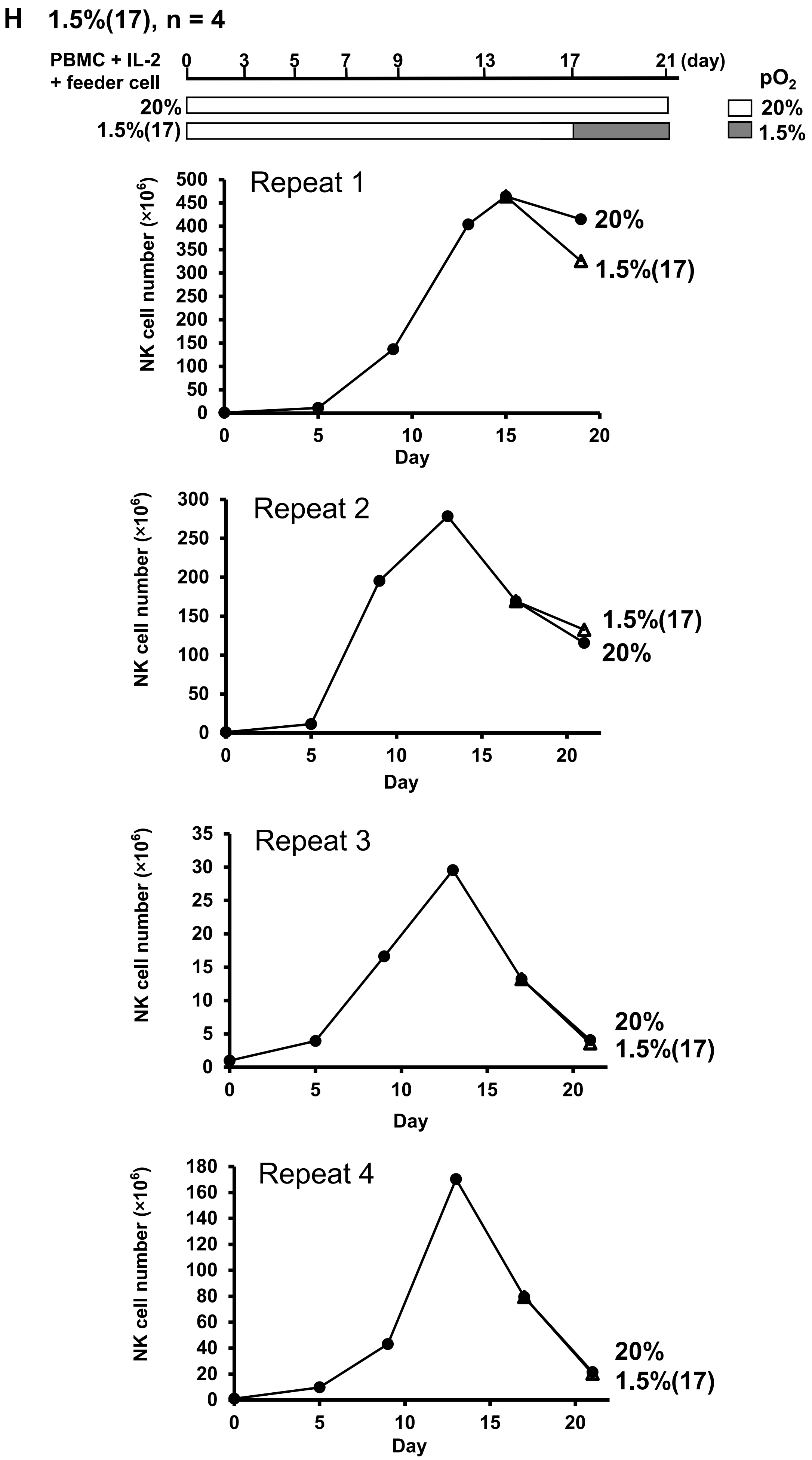

Figure S1H. Replicates of NK cell expansion under 20% and 1.5%(17) pO_2 conditions.

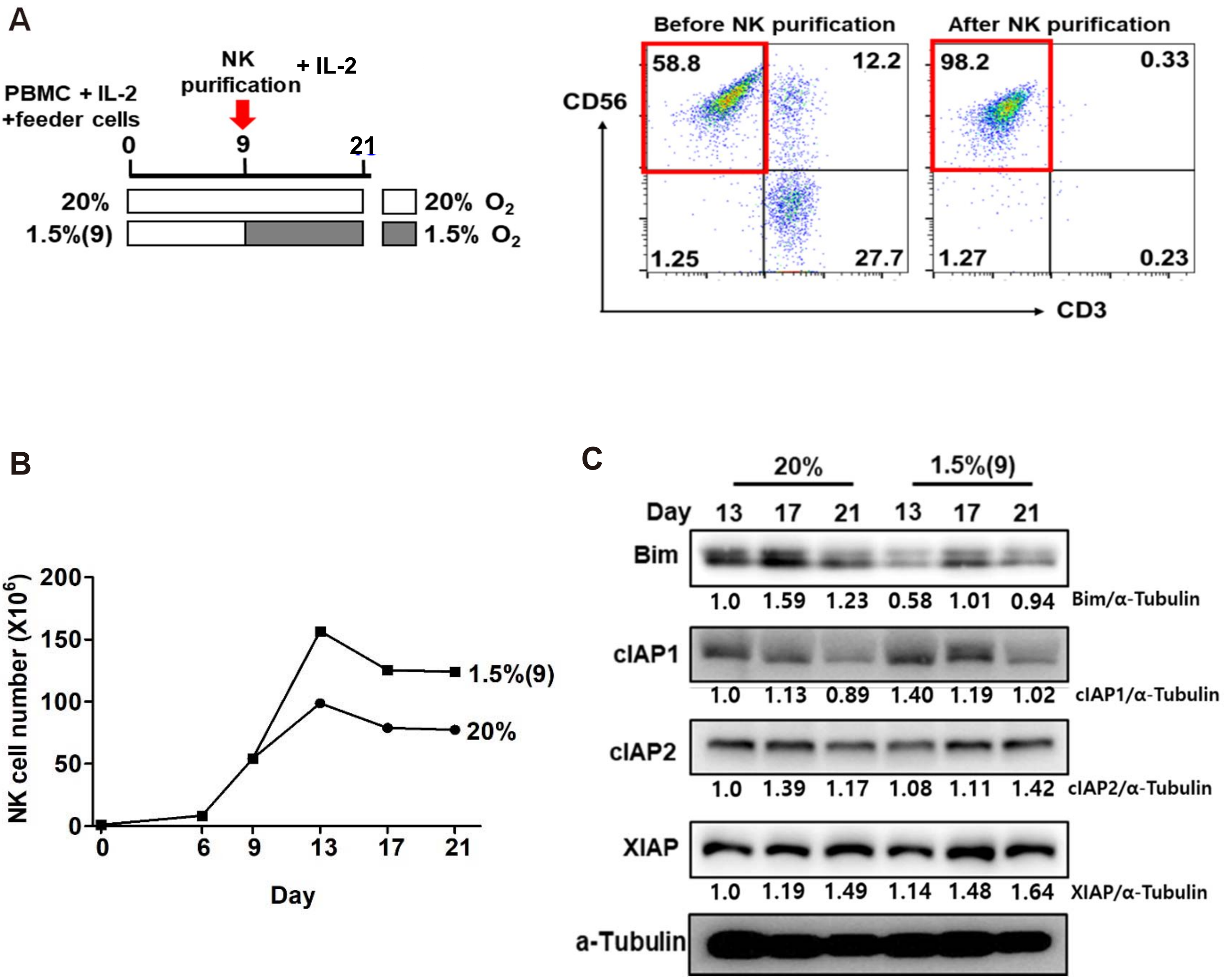

Figure S2. Highly purified NK cells from day 9 of culture showed enhanced proliferation in hypoxia-switched culture condition. (A) Schematic representation showing the date of NK cell purification (left) and the purity of NK cell after purification (right). (B) Representative growth curve of purified NK cells under normoxia and hypoxia. (C) Western blot analyses were performed to assess time-dependent changes of pro-apoptotic (Bim) and anti-apoptotic (cIAP1, cIAP2, and XIAP) marker expression in normoxic and hypoxic cultures of purified NK cells.

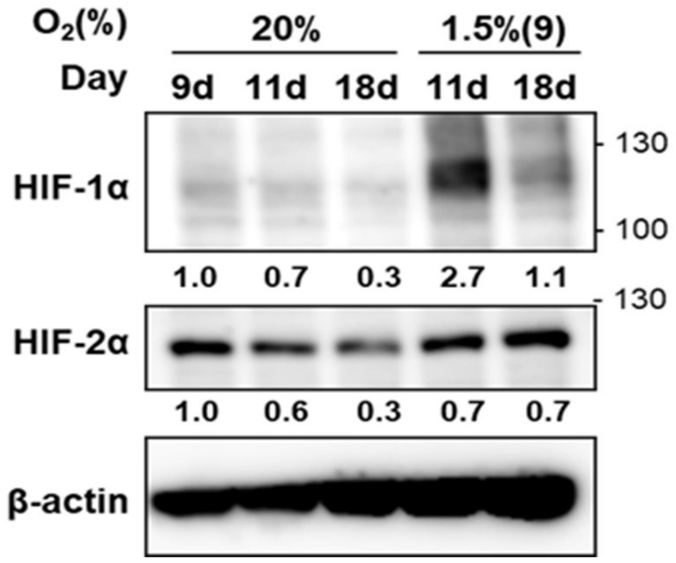

Figure S3. HIF-2α protein level was not significantly changed in the pre-activated hypoxic NK cells.

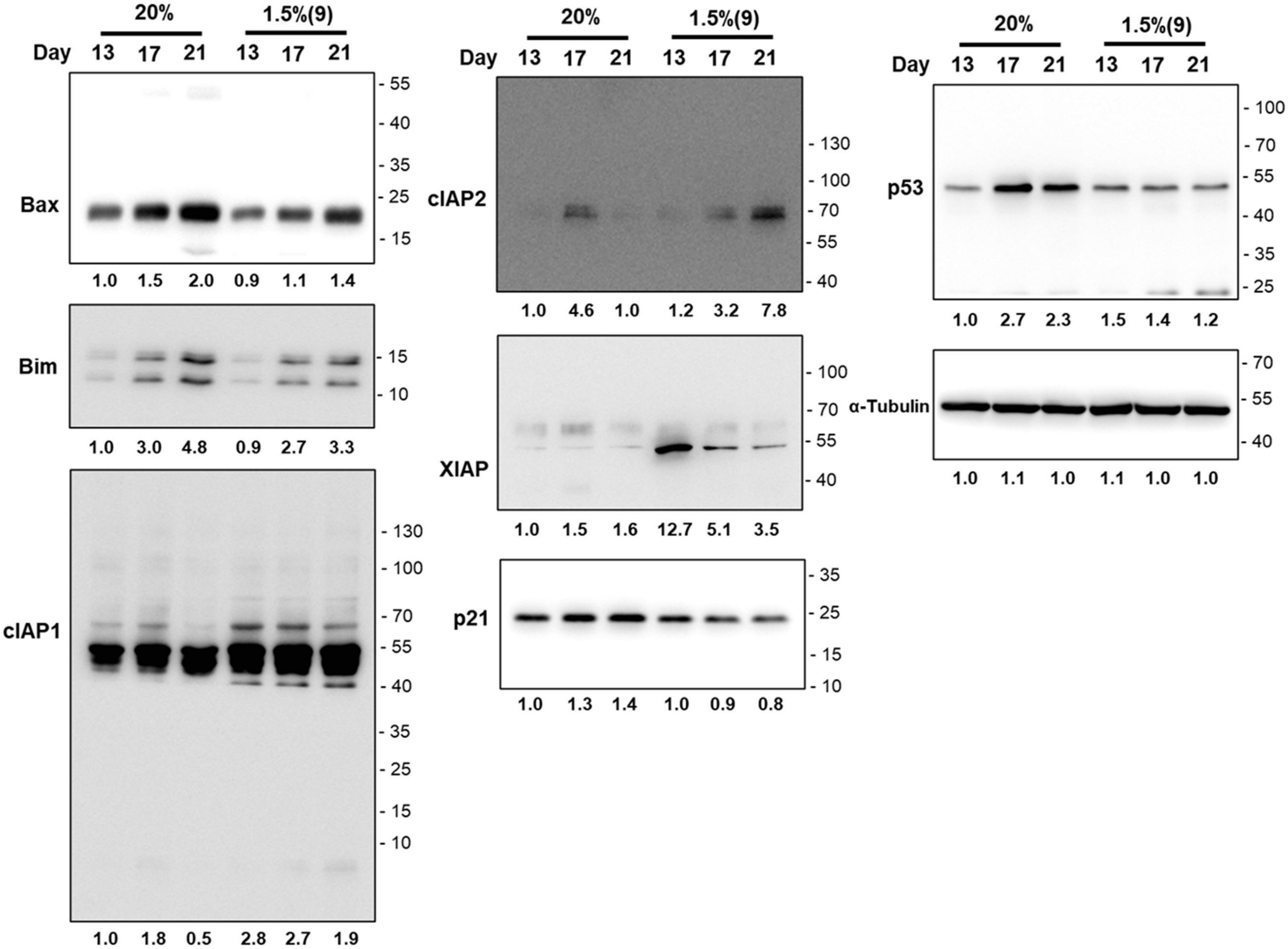

Figure S4. Original whole blots of western blot figures

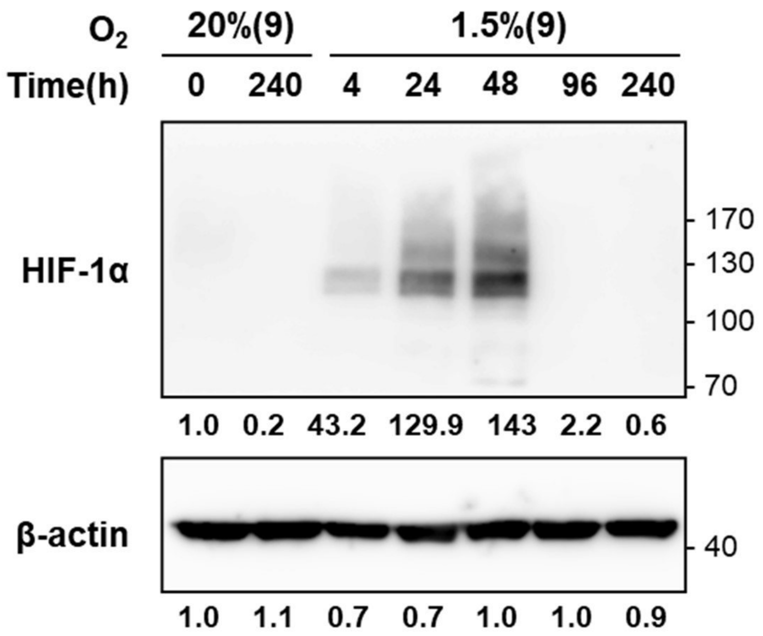

Figure S4. Original whole blots of western blot figures

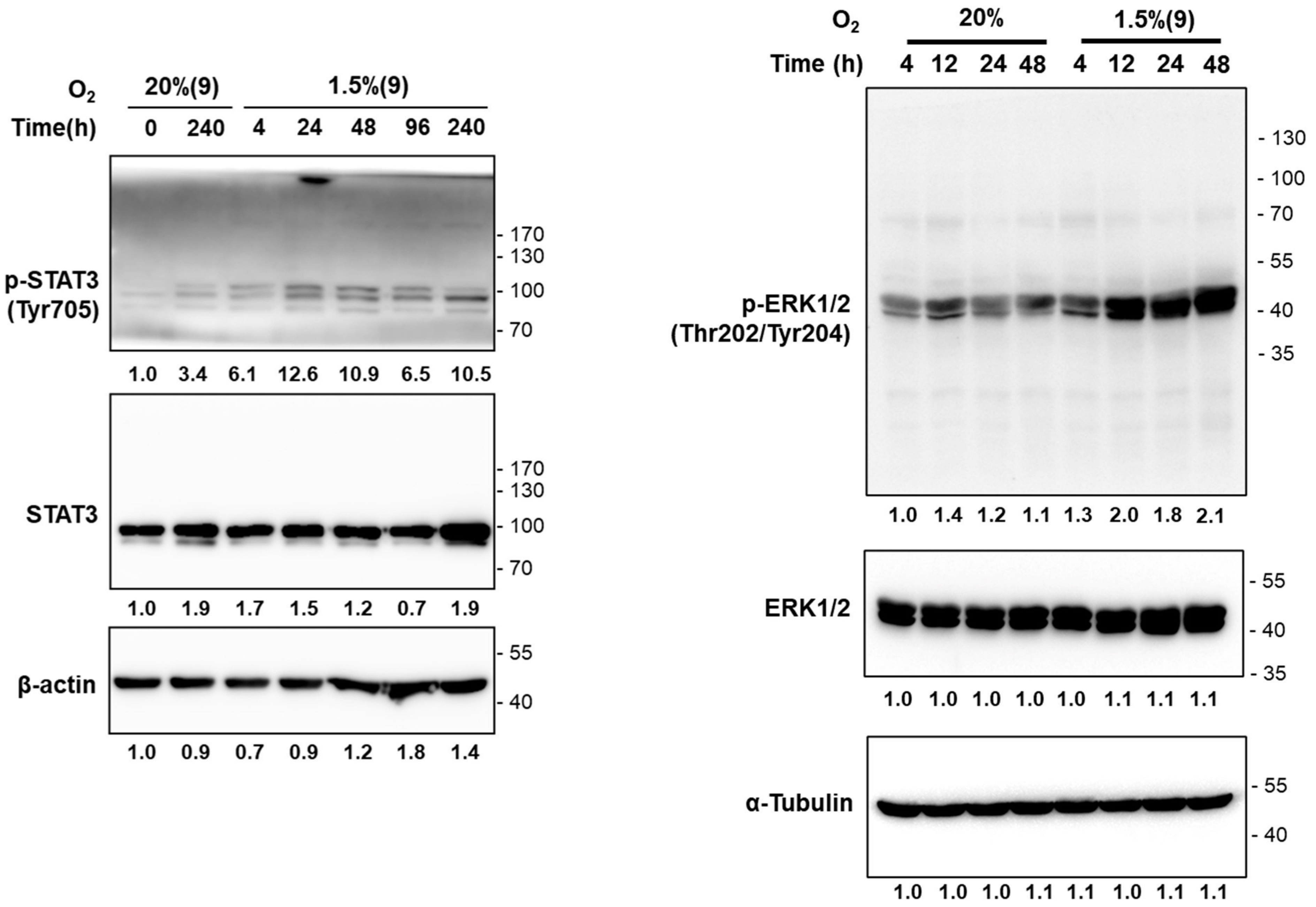

Figure S4. Original whole blots of western blot figures

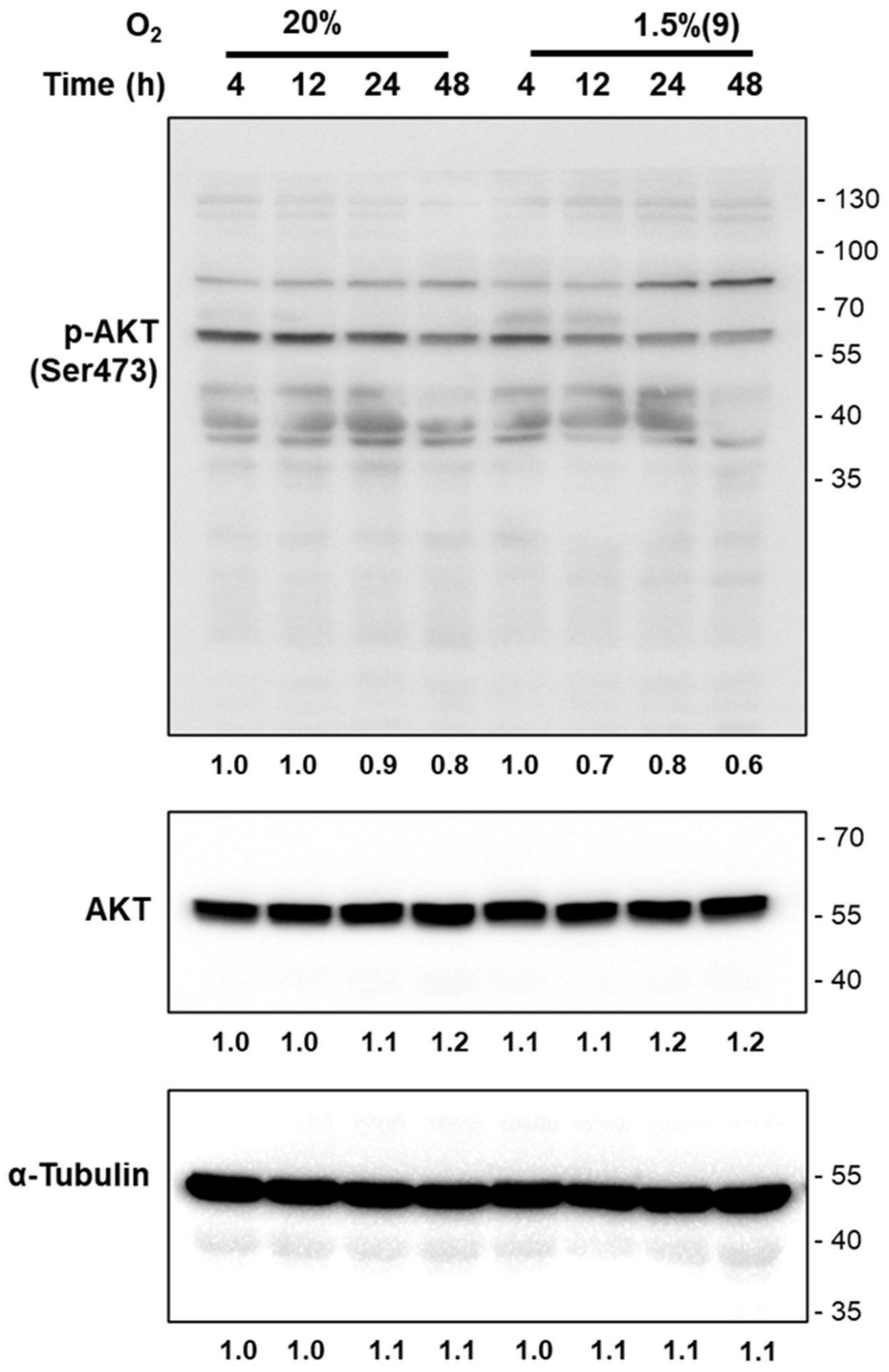

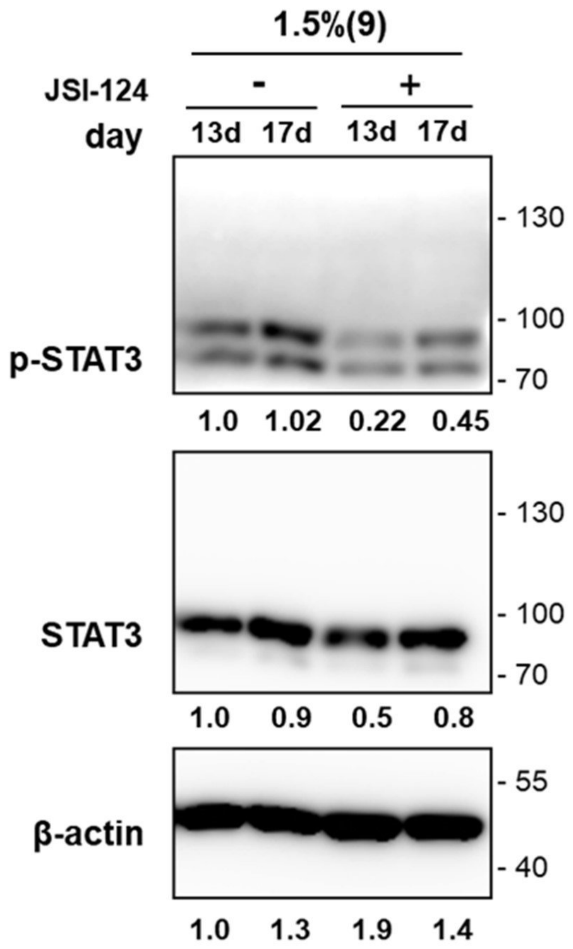

Figure S4. Original whole blots of western blot figures

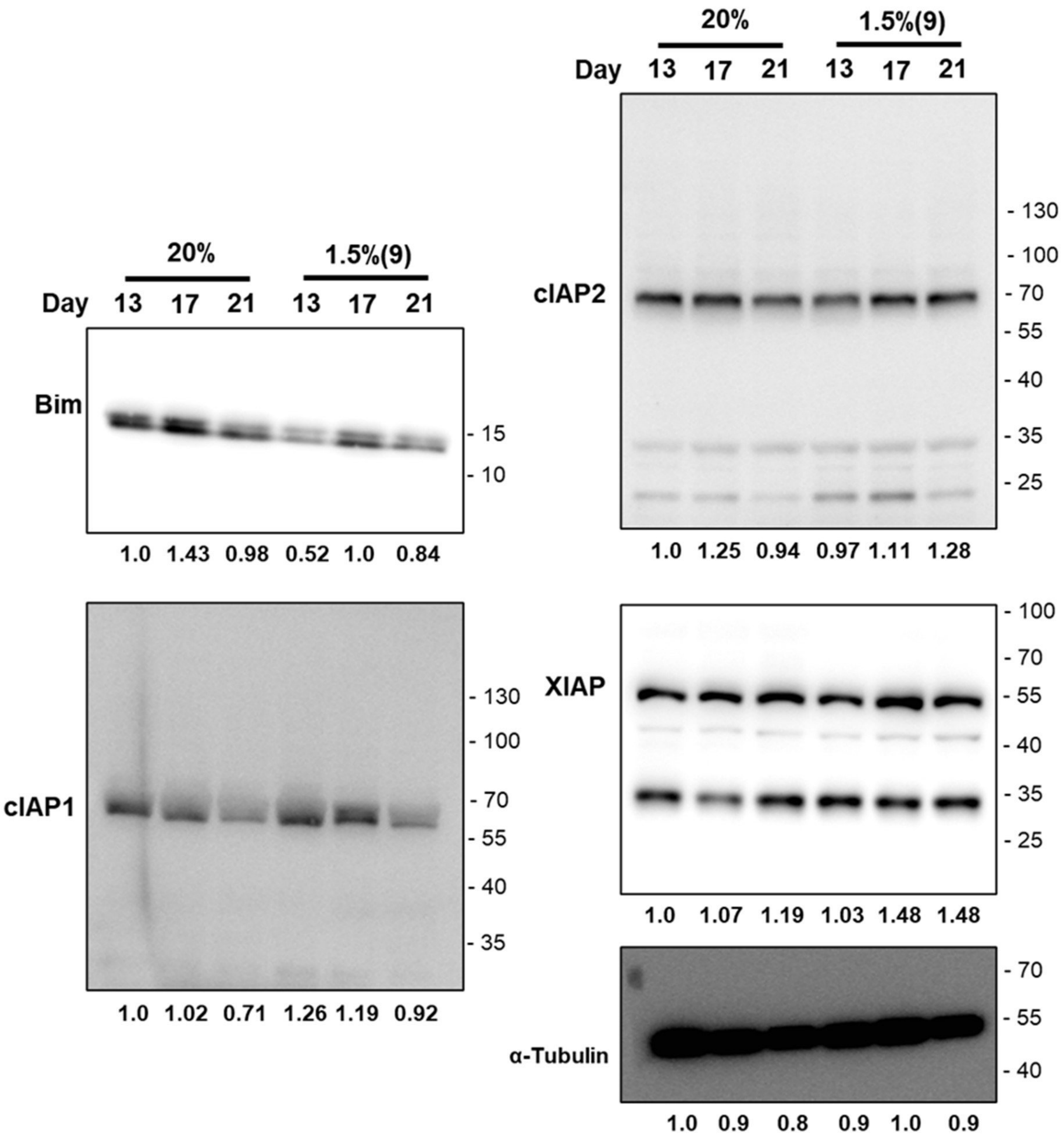

Figure S4. Original whole blots of western blot figures

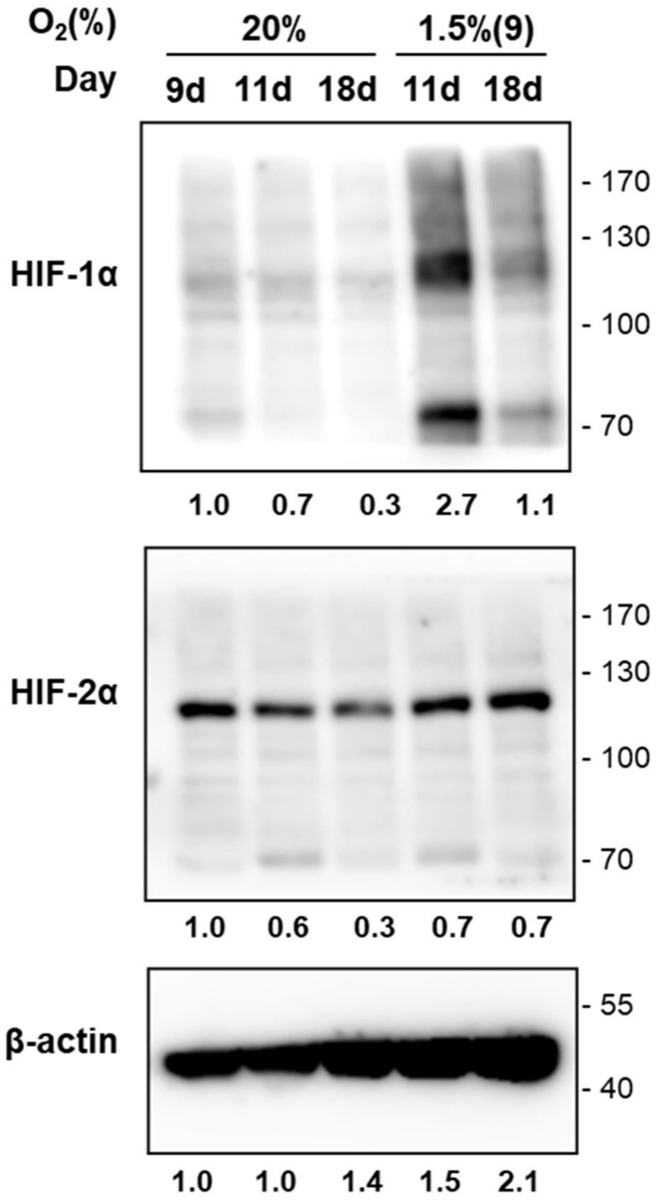

Figure S4. Original whole blots of western blot figures

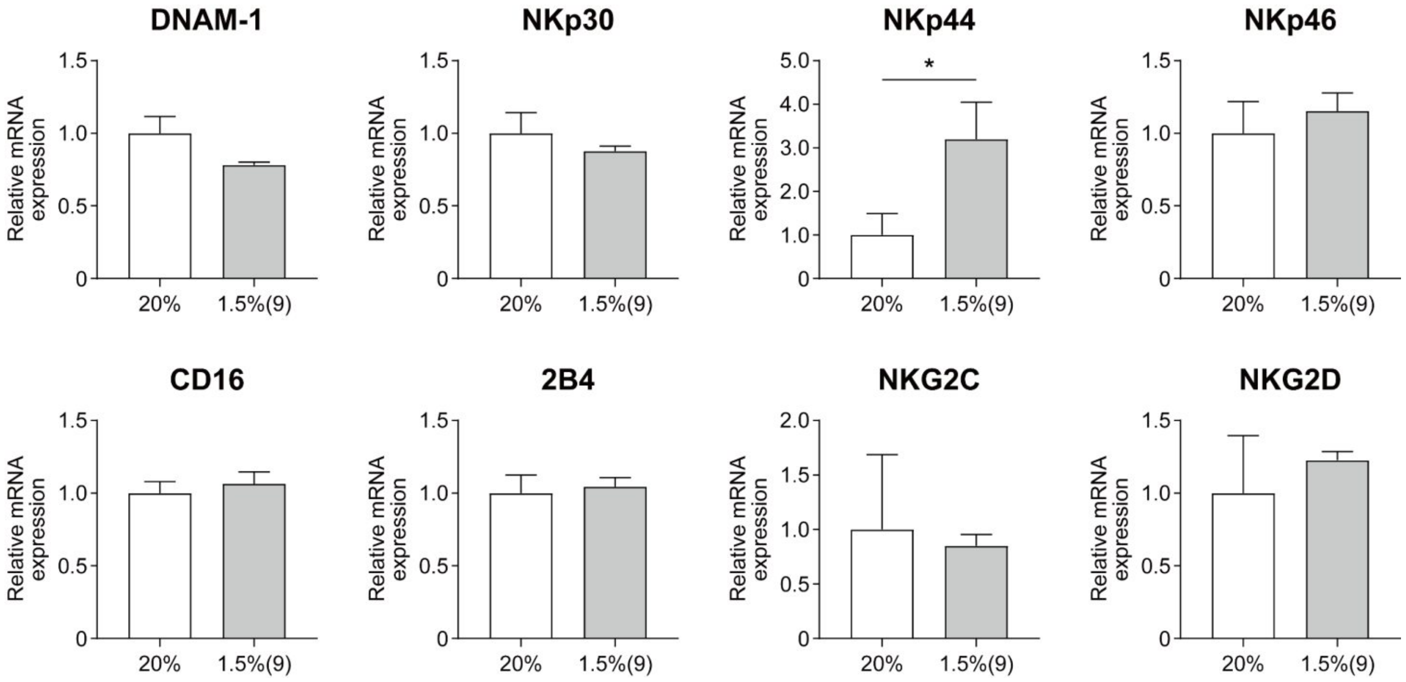

Figure S5. NKp44 is the only activating receptor upregulated at the level of mRNA in pre-activated hypoxic NK cells.

Table S1. 496 DEGs. For each DEG, entrez ID, gene symbol and description, log2-fold-change of mRNA expression levels between normoxic and pre-activated hypoxic NK cells are shown.

			mRNA expression (pre-activated hypoxic / Normoxic)		
EntrezID	Symbol	Description	Fold change (log2-value)	P-value	Up (1) /Down (-1)
205	AK4	adenylate kinase 4	6.360042142	0.000529015	1
3485	IGFBP2	insulin like growth factor binding protein 2	5.210921157	2.92E-05	1
8497	PPFIA4	PTPRF interacting protein alpha 4	4.656778878	5.84E-05	1
5210	PFKFB4	6-phosphofructo-2-kinase/fructose-2,6-biphosphatase 4	3.612089473	0.000625262	1
100506211	MIR210HG	MIR210 host gene	3.253683225	0.013554209	1
8614	STC2	stanniocalcin 2	3.083347013	0.004297118	1
3099	HK2	hexokinase 2	2.670853254	0.001684652	1
91947	ARRDC4	arrestin domain containing 4	2.41125841	0.000609862	1
7422	VEGFA	vascular endothelial growth factor A	2.34731109	0.032238918	1
284716	RIMKLA	ribosomal modification protein rimK like family member A	2.279499397	0.000196116	1
84419	C15orf48	chromosome 15 open reading frame 48	2.229148294	0.024960652	1
1015	CDH17	cadherin 17	2.150508556	0.000281266	1
112399	EGLN3	egl-9 family hypoxia inducible factor 3	1.974496274	0.000436392	1
161725	OTUD7A	OTU deubiquitinase 7A	1.956977263	0.031666421	1
91584	PLXNA4	plexin A4	1.930313824	0.000461303	1
6256	RXRA	retinoid X receptor alpha	1.924291057	0.005984034	1
55893	ZNF395	zinc finger protein 395	1.912063502	0.005152919	1
230	ALDOC	aldolase, fructose-bisphosphate C	1.903484244	0.000490064	1
4323	MMP14	matrix metallopeptidase 14	1.814576622	0.014839382	1
665	BNIP3L	BCL2 interacting protein 3 like	1.795390047	0.035393761	1
3892	KRT86	keratin 86	1.745543574	0.00146906	1
2118	ETV4	ETS variant 4	1.712835076	0.004167582	1
22918	CD93	CD93 molecule	1.69947027	0.039627696	1
4016	LOXL1	lysyl oxidase like 1	1.666155118	0.021290155	1
586	BCAT1	branched chain amino acid transaminase 1	1.641782551	0.014272547	1
9436	NCR2	natural cytotoxicity triggering receptor 2	1.624287321	0.032569326	1
3855	KRT7	keratin 7	1.594423553	0.003688388	1
5033	P4HA1	prolyl 4-hydroxylase subunit alpha 1	1.590528265	0.000894752	1
8974	P4HA2	prolyl 4-hydroxylase subunit alpha 2	1.566327533	0.007070373	1
79729	SH3D21	SH3 domain containing 21	1.559020361	0.019882466	1
146850	PIK3R6	phosphoinositide-3-kinase regulatory subunit 6	1.530229665	0.007054974	1
79369	B3GNT4	UDP-GlcNAc:betaGal beta-1,3-N-acetylglucosaminyltransferase 4	1.447121665	0.018202117	1
3887	KRT81	keratin 81	1.439699478	0.002289532	1
79166	LILRP2	leukocyte immunoglobulin-like receptor pseudogene 2	1.389162337	0.005508011	1
255877	BCL6B	B-cell CLL/lymphoma 6B	1.36708577	0.001519787	1
9783	RIMS3	regulating synaptic membrane exocytosis 3	1.348866772	0.014658891	1
23406	COTL1	coactosin like F-actin binding protein 1	1.300317898	0.02646504	1
3742	KCNA6	potassium voltage-gated channel subfamily A member 6	1.293992295	0.001769801	1
23254	KAZN	kazrin, periplakin interacting protein	1.286467324	0.001813508	1
730112	FAM166B	family with sequence similarity 166 member B	1.284324501	0.003225047	1
84632	AFAP1L2	actin filament associated protein 1 like 2	1.277806262	0.00189345	1
8701	DNAH11	dynein axonemal heavy chain 11	1.252618752	0.002812659	1
51517	NCKIPSD	NCK interacting protein with SH3 domain	1.24464972	0.02584476	1
57699	CPNE5	copine 5	1.235281608	0.002122176	1
2821	GPI	glucose-6-phosphate isomerase	1.230885094	0.002137576	1
284339	TMEM145	transmembrane protein 145	1.229983279	0.005237162	1
10848	PPP1R13L	protein phosphatase 1 regulatory subunit 13 like	1.222474563	0.002183321	1
26355	FAM162A	family with sequence similarity 162 member A	1.210298221	0.01977535	1
8291	DYSF	dysferlin	1.194106004	0.035723716	1
55534	MAML3	mastermind like transcriptional coactivator 3	1.191249072	0.002405933	1
5163	PDK1	pyruvate dehydrogenase kinase 1	1.183102264	0.003707637	1
25902	MTHFD1L	methylenetetrahydrofolate dehydrogenase (NADP+ dependent) 1-like	1.17437259	0.002507388	1
5230	PGK1	phosphoglycerate kinase 1	1.162256903	0.002562645	1
151056	PLB1	phospholipase B1	1.160937145	0.013528619	1
3939	LDHA	lactate dehydrogenase A	1.150687592	0.008563211	1
285598	ARL10	ADP ribosylation factor like GTPase 10	1.14074571	0.00390081	1
389114	ZNF662	zinc finger protein 662	1.139972011	0.002751967	1
60468	BACH2	BTB domain and CNC homolog 2	1.132098347	0.024738266	1
1238	ACKR2	atypical chemokine receptor 2	1.128196587	0.002805412	1
9315	NREP	neuronal regeneration related protein	1.125382448	0.002847081	1
10628	TXNIP	thioredoxin interacting protein	1.122995875	0.005318689	1
441168	FAM26F	family with sequence similarity 26 member F	1.098224117	0.036891355	1
57221	ARFGEF3	ARFGEF family member 3	1.09458367	0.005639586	1
3005	H1F0	H1 histone family member 0	1.058141132	0.003534847	1
387923	SERP2	stress associated endoplasmic reticulum protein family member 2	1.048846786	0.034349091	1
7167	TPI1	triosephosphate isomerase 1	1.048090685	0.00361592	1
79413	ZBED2	zinc finger BED-type containing 2	1.047484106	0.041594973	1
9079	LDB2	LIM domain binding 2	1.046829381	0.011554323	1
3687	ITGAX	integrin subunit alpha X	1.044750582	0.015757459	1
412	STS	steroid sulfatase	1.035974264	0.003767423	1
9123	SLC16A3	solute carrier family 16 member 3	1.034008464	0.003784182	1
2632	GBE1	1,4-alpha-glucan branching enzyme 1	1.027406457	0.003886542	1
5236	PGM1	phosphoglucomutase 1	1.025143307	0.012344675	1

4282	MIF	macrophage migration inhibitory factor (glycosylation-inhibiting factor)	0.991291129	0.00718836	1
55075	UACA	uveal autoantigen with coiled-coil domains and ankyrin repeats	0.981307468	0.004503199	1
11082	ESM1	endothelial cell specific molecule 1	0.981043816	0.004510446	1
8645	KCNK5	potassium two pore domain channel subfamily K member 5	0.979973192	0.043138538	1
1951	CELSR3	cadherin EGF LAG seven-pass G-type receptor 3	0.979369692	0.016435713	1
2023	ENO1	enolase 1	0.977778962	0.00458699	1
3479	IGF1	insulin like growth factor 1	0.975456359	0.038807904	1
92092	ZC3HAV1L	zinc finger CCCH-type containing, antiviral 1 like	0.973940375	0.01269705	1
26012	NSMF	NMDA receptor synaptonuclear signaling and neuronal migration factor	0.969560153	0.030727736	1
100431172	KLRF2	killer cell lectin like receptor F2	0.968409405	0.018773028	1
220992	ZNF485	zinc finger protein 485	0.953086747	0.004895658	1
89858	SIGLEC12	sialic acid binding Ig like lectin 12 (gene/pseudogene)	0.947233008	0.031950405	1
85458	DIXDC1	DIX domain containing 1	0.945695872	0.004991451	1
7436	VLDLR	very low density lipoprotein receptor	0.942567876	0.005024515	1
3805	KIR2DL4	killer cell immunoglobulin like receptor, two Ig domains and long cytoplasmic tail 4	0.934272815	0.01025828	1
10606	PAICS	phosphoribosylaminoimidazole carboxylase and phosphoribosylaminoimidazolesuccinocarboxamide synthase	0.921330685	0.005292419	1
23030	KDM4B	lysine demethylase 4B	0.9188927	0.005372134	1
158471	PRUNE2	prune homolog 2	0.918862099	0.005379381	1
10570	DPYSL4	dihydropyrimidinase like 4	0.917578973	0.005393648	1
84951	TNS4	tensin 4	0.906000061	0.006245825	1
23036	ZNF292	zinc finger protein 292	0.903363198	0.019563155	1
5315	PKM	pyruvate kinase, muscle	0.898250496	0.040358716	1
29015	SLC43A3	solute carrier family 43 member 3	0.895809834	0.005770934	1
56833	SLAMF8	SLAM family member 8	0.891267199	0.005908623	1
55423	SIRPG	signal regulatory protein gamma	0.887909976	0.005947574	1
22915	MMRN1	multimerin 1	0.880306648	0.006133499	1
1293	COL6A3	collagen type VI alpha 3 chain	0.87420891	0.006261224	1
226	ALDOA	aldolase, fructose-bisphosphate A	0.872745446	0.006294514	1
199	AIF1	allograft inflammatory factor 1	0.870443452	0.00636132	1
4923	NTSR1	neurotensin receptor 1	0.870038021	0.033873747	1
55552	ZNF823	zinc finger protein 823	0.867914642	0.037027006	1
7023	TFAP4	transcription factor AP-4	0.848428754	0.023647399	1
284422	SMIM24	small integral membrane protein 24	0.846478906	0.034097718	1
387357	THEMIS	thymocyte selection associated	0.843637119	0.006924758	1
3681	ITGAD	integrin subunit alpha D	0.82178331	0.011640605	1
7772	ZNF229	zinc finger protein 229	0.817159327	0.007517183	1
5831	PYCR1	pyrroline-5-carboxylate reductase 1	0.816896076	0.007533941	1
4234	METTL1	methyltransferase like 1	0.814255366	0.009650229	1
2736	GLI2	GLI family zinc finger 2	0.813875731	0.045031082	1
113235	SLC46A1	solute carrier family 46 member 1	0.813342708	0.007593953	1
133121	ENPP6	ectonucleotide pyrophosphatase/phosphodiesterase 6	0.809727504	0.039877031	1
162515	SLC16A11	solute carrier family 16 member 11	0.809307188	0.026496745	1
10112	KIF20A	kinesin family member 20A	0.80682788	0.034831003	1
5028	P2RY1	purinergic receptor P2Y1	0.80567488	0.00880077	1
1615	DARS	aspartyl-tRNA synthetase	0.804549366	0.007868199	1
57194	ATP10A	ATPase phospholipid transporting 10A (putative)	0.799120018	0.008037366	1
6624	FSCN1	fascin actin-bundling protein 1	0.792337638	0.008345581	1
8482	SEMA7A	semaphorin 7A (John Milton Hagen blood group)	0.787495737	0.008459945	1
81610	FAM83D	family with sequence similarity 83 member D	0.779291794	0.00869388	1
80381	CD276	CD276 molecule	0.765663339	0.016952047	1
7567	ZNF19	zinc finger protein 19	0.765085102	0.035505633	1
2030	SLC29A1	solute carrier family 29 member 1 (Augustine blood group)	0.763605931	0.009335221	1
84319	CMSS1	cms1 ribosomal small subunit homolog (yeast)	0.762894633	0.009373493	1
51209	RAB9B	RAB9B, member RAS oncogene family	0.762024357	0.009432599	1
79850	FAM57A	family with sequence similarity 57 member A	0.746891166	0.009910887	1
26150	RIBC2	RIB43A domain with coiled-coils 2	0.741413476	0.040795788	1
5818	NECTIN1	nectin cell adhesion molecule 1	0.732626328	0.010365623	1
26509	MYOF	myoferlin	0.732145139	0.010391893	1
79865	TREML2	triggering receptor expressed on myeloid cells like 2	0.726660985	0.010623563	1
8996	NOL3	nucleolar protein 3	0.724290016	0.010720716	1
321	APBA2	amyloid beta precursor protein binding family A member 2	0.720956375	0.010885127	1
2653	GCSH	glycine cleavage system protein H	0.716790432	0.011056785	1
991	CDC20	cell division cycle 20	0.716202185	0.026716639	1
28514	DLL1	delta like canonical Notch ligand 1	0.71389073	0.012033063	1
7465	WEE1	WEE1 G2 checkpoint kinase	0.712409264	0.011315632	1
5347	PLK1	polo like kinase 1	0.709435541	0.033492159	1
171389	NLRP6	NLR family pyrin domain containing 6	0.708263673	0.011488649	1
125061	AFMID	arylformamidase	0.705998481	0.04633256	1
1462	VCAN	versican	0.704974657	0.04705203	1
84627	ZNF469	zinc finger protein 469	0.703901948	0.011699032	1
57336	ZNF287	zinc finger protein 287	0.703686171	0.011706279	1
339500	ZNF678	zinc finger protein 678	0.70227649	0.023447659	1
83903	GSG2	germ cell associated 2, haspin	0.697475626	0.011982109	1
5451	POU2F1	POU class 2 homeobox 1	0.694536817	0.012142671	1
23543	RBFOX2	RNA binding protein, fox-1 homolog 2	0.693782756	0.032776312	1
27033	ZBTB32	zinc finger and BTB domain containing 32	0.692159258	0.012233935	1
50636	ANO7	anoctamin 7	0.690524236	0.01733907	1

51805	COQ3	coenzyme Q3, methyltransferase	0.689915194	0.012327917	1
162387	MFSD6L	major facilitator superfamily domain containing 6 like	0.687423057	0.012449074	1
79816	TLE6	transducin like enhancer of split 6	0.685413428	0.030165204	1
170958	ZNF525	zinc finger protein 525	0.684606443	0.012565702	1
400506	KNOP1	lysine rich nucleolar protein 1	0.68443904	0.016459265	1
692086	SNORD17	small nucleolar RNA, C/D box 17	0.683358509	0.012628659	1
4998	ORC1	origin recognition complex subunit 1	0.682839339	0.012689804	1
29800	ZDHHC1	zinc finger DHHC-type containing 1	0.682403507	0.012704297	1
990	CDC6	cell division cycle 6	0.681769706	0.012727849	1
5214	PFKP	phosphofructokinase, platelet	0.681428269	0.01276046	1
4744	NEFH	neurofilament heavy	0.680772305	0.04126162	1
5198	PFAS	phosphoribosylformylglycinamidine synthase	0.679753289	0.044957255	1
100506178	LOC100506178	uncharacterized LOC100506178	0.679585089	0.013795391	1
51659	GINS2	GINS complex subunit 2	0.6786833	0.012879579	1
57190	SELENON	selenoprotein N	0.677197614	0.03718032	1
84904	ARHGEF39	Rho guanine nucleotide exchange factor 39	0.674323189	0.013089056	1
11045	UPK1A	uroplakin 1A	0.670598059	0.030296779	1
3581	IL9R	interleukin 9 receptor	0.667599984	0.035157108	1
2597	GAPDH	glyceraldehyde-3-phosphate dehydrogenase	0.666764471	0.013474721	1
30832	ZNF354C	zinc finger protein 354C	0.663394352	0.014435373	1
150726	FBXO41	F-box protein 41	0.662998237	0.01359769	1
51154	MRTO4	MRT4 homolog, ribosome maturation factor	0.661670513	0.013647965	1
155038	GIMAP8	GTPase, IMAP family member 8	0.661536799	0.013665402	1
5158	PDE6B	phosphodiesterase 6B	0.658150789	0.016834739	1
342892	ZNF850	zinc finger protein 850	0.655903346	0.013898885	1
84969	TOX2	TOX high mobility group box family member 2	0.655694095	0.02744698	1
2026	ENO2	enolase 2	0.655472856	0.013939874	1
284307	ZIK1	zinc finger protein interacting with K protein 1	0.654952409	0.049472004	1
254050	LRRC43	leucine rich repeat containing 43	0.654623252	0.033048293	1
3241	HPCAL1	hippocalcin like 1	0.653919632	0.013975429	1
56242	ZNF253	zinc finger protein 253	0.652983246	0.015013984	1
1306	COL15A1	collagen type XV alpha 1 chain	0.646612462	0.014356565	1
126068	ZNF441	zinc finger protein 441	0.646001612	0.014428127	1
9249	DHRS3	dehydrogenase/reductase 3	0.645883756	0.01444262	1
7762	ZNF215	zinc finger protein 215	0.645827318	0.014449867	1
728084	LOC728084	uncharacterized LOC728084	0.64580269	0.014465266	1
701	BUB1B	BUB1 mitotic checkpoint serine/threonine kinase B	0.64498751	0.021709336	1
100533107	RTEL1-TNFRSF6B	RTEL1-TNFRSF6B readthrough (NMD candidate)	0.644592327	0.014528223	1
899	CCNF	cyclin F	0.643175679	0.027728698	1
3077	HFE	hemochromatosis	0.64077813	0.014787975	1
2187	FANCB	Fanconi anemia complementation group B	0.63902734	0.045846119	1
158405	KIAA1958	KIAA1958	0.635663314	0.015197871	1
117144	CATSPER1	cation channel sperm associated 1	0.63551107	0.015225273	1
122704	MRPL52	mitochondrial ribosomal protein L52	0.634293579	0.015363415	1
3615	IMPDH2	inosine monophosphate dehydrogenase 2	0.631879197	0.015504048	1
23017	FAIM2	Fas apoptotic inhibitory molecule 2	0.630722065	0.015560664	1
651302	ZNF192P1	zinc finger protein 192 pseudogene 1	0.629883878	0.015688841	1
6856	SYPL1	synaptophysin like 1	0.629658479	0.037829587	1
898	CCNE1	cyclin E1	0.629392931	0.018393025	1
100129792	CCDC152	coiled-coil domain containing 152	0.626233476	0.015987771	1
126231	ZNF573	zinc finger protein 573	0.624480475	0.016072921	1
9319	TRIP13	thyroid hormone receptor interactor 13	0.624249427	0.022417483	1
6573	SLC19A1	solute carrier family 19 member 1	0.622519354	0.016237785	1
55789	DEPDC1B	DEP domain containing 1B	0.621783189	0.048090358	1
64127	NOD2	nucleotide binding oligomerization domain containing 2	0.61956485	0.016428466	1
388559	ZNF888	zinc finger protein 888	0.618390044	0.016540565	1
124222	PAQR4	progestin and adipoQ receptor family member 4	0.617495731	0.036746646	1
285605	DTWD2	DTW domain containing 2	0.616708477	0.01665357	1
100128191	TMPO-AS1	TMPO antisense RNA 1	0.616071484	0.016704977	1
112755	STX1B	syntaxin 1B	0.612093729	0.016974693	1
54475	NLE1	notchless homolog 1	0.611885075	0.01698194	1
83449	PMFBP1	polyamine modulated factor 1 binding protein 1	0.610669819	0.017072298	1
4500	MT1L	metallothionein 1L, pseudogene	0.609802863	0.029774331	1
8801	SUCLG2	succinate-CoA ligase GDP-forming beta subunit	0.608363322	0.017219951	1
79733	E2F8	E2F transcription factor 8	0.608301058	0.032713809	1
100128881	VPS9D1-AS1	VPS9D1 antisense RNA 1	0.608071413	0.017235351	1
2669	GEM	GTP binding protein overexpressed in skeletal muscle	0.604351902	0.029274755	1
29104	N6AMT1	N-6 adenine-specific DNA methyltransferase 1 (putative)	0.602012943	0.017682387	1
3812	KIR3DL2	killer cell immunoglobulin like receptor, three Ig domains and long cytoplasmic tail 2	0.600306409	0.017886882	1
54583	EGLN1	egl-9 family hypoxia inducible factor 1	0.599383848	0.017923116	1
90233	ZNF551	zinc finger protein 551	0.597226438	0.018060805	1
152002	XXYLT1	xyloside xylosyltransferase 1	0.596539598	0.018107456	1
100137047	JMJD7	jumonji domain containing 7	0.595029271	0.04310706	1
7374	UNG	uracil DNA glycosylase	0.594503927	0.018242881	1
152579	SCFD2	sec1 family domain containing 2	0.593738564	0.018635792	1
79000	AUNIP	aurora kinase A and ninein interacting protein	0.592570929	0.031289135	1
219	ALDH1B1	aldehyde dehydrogenase 1 family member B1	0.592429912	0.0183543	1

55226	NAT10	N-acetyltransferase 10	0.591947912	0.018407292	1
64858	DCLRE1B	DNA cross-link repair 1B	0.591493611	0.018465719	1
9381	OTOF	otoferlin	0.591017485	0.030244466	1
2583	B4GALNT1	beta-1,4-N-acetyl-galactosaminyltransferase 1	0.589320296	0.018610882	1
79915	ATAD5	ATPase family, AAA domain containing 5	0.589025866	0.018643039	1
55888	ZKSCAN7	zinc finger with KRAB and SCAN domains 7	0.588567866	0.042640548	1
89978	DPH6	diphthamine biosynthesis 6	0.588405639	0.018730227	1
153768	PRELID2	PRELI domain containing 2	0.587423949	0.029009794	1
580	BARD1	BRCA1 associated RING domain 1	0.584014061	0.019179075	1
55711	FAR2	fatty acyl-CoA reductase 2	0.583260056	0.027012399	1
27340	UTP20	UTP20, small subunit processome component	0.582261122	0.01934643	1
54938	SARS2	seryl-tRNA synthetase 2, mitochondrial	0.580148172	0.019455585	1
113178	SCAMP4	secretory carrier membrane protein 4	-0.581376032	0.019477778	-1
3983	ABLIM1	actin binding LIM protein 1	-0.583708847	0.019280756	-1
411	ARSB	arylsulfatase B	-0.583783717	0.019263998	-1
130589	GALM	galactose mutarotase	-0.584144897	0.044995527	-1
105374104	LINC02021	long intergenic non-protein coding RNA 2021	-0.585661212	0.038059446	-1
11145	PLA2G16	phospholipase A2 group XVI	-0.58601494	0.019032101	-1
5837	PYGM	glycogen phosphorylase, muscle associated	-0.586383786	0.019016475	-1
290	ANPEP	alanyl aminopeptidase, membrane	-0.588344714	0.018812659	-1
950	SCARB2	scavenger receptor class B member 2	-0.588863091	0.01872366	-1
2288	FKBP4	FK506 binding protein 4	-0.589908279	0.023222782	-1
80737	VWA7	von Willebrand factor A domain containing 7	-0.590175329	0.01860907	-1
5184	PEPD	peptidase D	-0.590349122	0.018592312	-1
152485	ZNF827	zinc finger protein 827	-0.59194314	0.02249765	-1
79735	TBC1D17	TBC1 domain family member 17	-0.594230925	0.018281379	-1
22933	SIRT2	sirtuin 2	-0.597289269	0.01806307	-1
9588	PRDX6	peroxiredoxin 6	-0.600771005	0.017828681	-1
10561	IFI44	interferon induced protein 44	-0.600872385	0.017811923	-1
5252	PHF1	PHD finger protein 1	-0.601506693	0.017749193	-1
79567	FAM65A	family with sequence similarity 65 member A	-0.602183137	0.017688954	-1
9658	ZNF516	zinc finger protein 516	-0.605395453	0.017478118	-1
100129550	LINC02035	long intergenic non-protein coding RNA 2035	-0.60540187	0.017470871	-1
57282	SLC4A10	solute carrier family 4 member 10	-0.606013793	0.017436902	-1
113622	ADPRHL1	ADP-ribosylhydrolase like 1	-0.607338986	0.017347449	-1
114881	OSBPL7	oxysterol binding protein like 7	-0.60745646	0.017340203	-1
91523	PCED1B	PC-esterase domain containing 1B	-0.607929608	0.028310706	-1
4193	MDM2	MDM2 proto-oncogene	-0.608792359	0.020728755	-1
347902	AMIGO2	adhesion molecule with Ig like domain 2	-0.609623292	0.017182812	-1
613037	NPIPB13	nuclear pore complex interacting protein family, member B13	-0.613761572	0.01689294	-1
85462	FHDC1	FH2 domain containing 1	-0.615107725	0.016776991	-1
3656	IRAK2	interleukin 1 receptor associated kinase 2	-0.615911681	0.016742569	-1
9693	RAPGEF2	Rap guanine nucleotide exchange factor 2	-0.620367519	0.016389062	-1
78999	LRFN4	leucine rich repeat and fibronectin type III domain containing 4	-0.622455549	0.016269037	-1
8878	SQSTM1	sequestosome 1	-0.622969322	0.01618819	-1
29851	ICOS	inducible T-cell costimulator	-0.623719371	0.016170752	-1
353189	SLCO4C1	solute carrier organic anion transporter family member 4C1	-0.623937127	0.016153994	-1
4086	SMAD1	SMAD family member 1	-0.625729362	0.039479364	-1
581	BAX	BCL2 associated X, apoptosis regulator	-0.625987293	0.016042575	-1
2166	FAAH	fatty acid amide hydrolase	-0.626494127	0.015999547	-1
1649	DDIT3	DNA damage inducible transcript 3	-0.626587201	0.015973504	-1
351	APP	amyloid beta precursor protein	-0.62688762	0.015966257	-1
157506	RDH10	retinol dehydrogenase 10	-0.629762832	0.01865323	-1
10389	SCML2	sex comb on midleg-like 2 (Drosophila)	-0.630873974	0.027900357	-1
10957	PNRC1	proline rich nuclear receptor coactivator 1	-0.633869978	0.031600521	-1
10608	MXD4	MAX dimerization protein 4	-0.636120581	0.015190172	-1
56995	TULP4	tubby like protein 4	-0.638618967	0.014997679	-1
5101	PCDH9	protocadherin 9	-0.638980861	0.039299326	-1
23555	TSPAN15	tetraspanin 15	-0.643233468	0.014690143	-1
5168	ENPP2	ectonucleotide pyrophosphatase/phosphodiesterase 2	-0.644241224	0.014640548	-1
64398	MPP5	membrane palmitoylated protein 5	-0.645183907	0.042693087	-1
79006	METRN	meteorin, glial cell differentiation regulator	-0.645686819	0.034291343	-1
9811	CTIF	cap binding complex dependent translation initiation factor	-0.646400005	0.014474098	-1
307	ANXA4	annexin A4	-0.647394503	0.048376833	-1
10903	MTMR11	myotubularin related protein 11	-0.648004429	0.024453604	-1
83719	YPEL3	yippee like 3	-0.648266274	0.014340939	-1
22882	ZHX2	zinc fingers and homeoboxes 2	-0.650463081	0.014237219	-1
100188949	LINC00426	long intergenic non-protein coding RNA 426	-0.651283708	0.014195324	-1
645432	ARRDC5	arrestin domain containing 5	-0.651353543	0.014188077	-1
2549	GAB1	GRB2 associated binding protein 1	-0.651434593	0.01418083	-1
9636	ISG15	ISG15 ubiquitin-like modifier	-0.651992566	0.014116515	-1
284252	KCTD1	potassium channel tetramerization domain containing 1	-0.654898716	0.02431365	-1
285638	LOC285638	uncharacterized LOC285638	-0.657818703	0.013889373	-1
23022	PALLD	palladin, cytoskeletal associated protein	-0.660249486	0.01376414	-1
9540	TP53I3	tumor protein p53 inducible protein 3	-0.663451003	0.038716639	-1
100505622	LOC100505622	uncharacterized LOC100505622	-0.664921524	0.013562136	-1
9467	SH3BP5	SH3 domain binding protein 5	-0.667054733	0.0134908	-1

1026	CDKN1A	cyclin dependent kinase inhibitor 1A	-0.668916427	0.013380513	-1
55959	SULF2	sulfatase 2	-0.672633612	0.013208175	-1
6528	SLC5A5	solute carrier family 5 member 5	-0.672747491	0.013200928	-1
11046	SLC35D2	solute carrier family 35 member D2	-0.673162194	0.013193682	-1
114793	FMNL2	formin like 2	-0.674397256	0.01314001	-1
3017	HIST1H2BD	histone cluster 1 H2B family member d	-0.675434277	0.013094038	-1
283229	CRACR2B	calcium release activated channel regulator 2B	-0.676275512	0.013052369	-1
25939	SAMHD1	SAM and HD domain containing deoxynucleoside triphosphate triphosphohydrolase 1	-0.679460021	0.012902451	-1
85236	HIST1H2BK	histone cluster 1 H2B family member k	-0.68061739	0.012857612	-1
2034	EPAS1	endothelial PAS domain protein 1	-0.685440556	0.026652098	-1
64121	RRAGC	Ras related GTP binding C	-0.686961031	0.012502746	-1
93953	GCNA	germ cell nuclear acidic peptidase	-0.688572294	0.039734586	-1
201895	SMIM14	small integral membrane protein 14	-0.691427782	0.012307083	-1
64342	HS1BP3	HCLS1 binding protein 3	-0.692604077	0.048180264	-1
115	ADCY9	adenylate cyclase 9	-0.699709572	0.01736534	-1
10046	MAMLD1	mastermind like domain containing 1	-0.699933829	0.011900357	-1
10160	FARP1	FERM, ARH/RhoGEF and pleckstrin domain protein 1	-0.702107018	0.011832192	-1
112483	SAT2	spermidine/spermine N1-acetyltransferase family member 2	-0.703049178	0.01209919	-1
3655	ITGA6	integrin subunit alpha 6	-0.705285692	0.031242258	-1
5243	ABCB1	ATP binding cassette subfamily B member 1	-0.705359689	0.023280303	-1
254428	SLC41A1	solute carrier family 41 member 1	-0.707743791	0.011533488	-1
5476	CTSA	cathepsin A	-0.709976774	0.037301704	-1
23529	CLCF1	cardiotrophin-like cytokine factor 1	-0.716004219	0.01112495	-1
54749	EPDR1	ependymin related 1	-0.716211459	0.011117704	-1
9435	CHST2	carbohydrate sulfotransferase 2	-0.716738291	0.011090755	-1
9314	KLF4	Kruppel like factor 4	-0.720087222	0.010961218	-1
151742	PPM1L	protein phosphatase, Mg2+/Mn2+ dependent 1L	-0.72179162	0.010878333	-1
719	C3AR1	complement C3a receptor 1	-0.722028417	0.033849289	-1
291	SLC25A4	solute carrier family 25 member 4	-0.723640121	0.010773481	-1
221184	CPNE2	copine 2	-0.724121926	0.031330578	-1
407006	MIR221	microRNA 221	-0.724193486	0.011293891	-1
11147	HHLA3	HERV-H LTR-associating 3	-0.726459882	0.010666818	-1
8531	YBX3	Y-box binding protein 3	-0.72801118	0.010576006	-1
100132948	FAM27C	family with sequence similarity 27 member C	-0.728883198	0.023010361	-1
5341	PLEK	pleckstrin	-0.737791098	0.024084244	-1
54843	SYTL2	synaptotagmin like 2	-0.742047348	0.046728642	-1
59271	EVA1C	eva-1 homolog C	-0.742053122	0.010133726	-1
718	C3	complement C3	-0.742065555	0.010126479	-1
9619	ABCG1	ATP binding cassette subfamily G member 1	-0.742171807	0.010119232	-1
10129	FRY	FRY microtubule binding protein	-0.742765776	0.010094321	-1
5366	PMAIP1	phorbol-12-myristate-13-acetate-induced protein 1	-0.743432834	0.018175848	-1
2781	GNAZ	G protein subunit alpha z	-0.745441062	0.01005152	-1
388403	YPEL2	yippee like 2	-0.750430045	0.044493008	-1
4163	MCC	mutated in colorectal cancers	-0.751849922	0.046126479	-1
9771	RAPGEF5	Rap guanine nucleotide exchange factor 5	-0.752788422	0.009786786	-1
100505576	LINC00672	long intergenic non-protein coding RNA 672	-0.755044422	0.009714997	-1
3162	HMOX1	heme oxygenase 1	-0.755362362	0.009674687	-1
85478	CCDC65	coiled-coil domain containing 65	-0.755882217	0.009659288	-1
85012	TCEAL3	transcription elongation factor A like 3	-0.762311146	0.009509143	-1
165215	FAM171B	family with sequence similarity 171 member B	-0.762674413	0.009486724	-1
64782	AEN	apoptosis enhancing nuclease	-0.763228745	0.009459322	-1
3824	KLRD1	killer cell lectin like receptor D1	-0.763984393	0.009405877	-1
90990	KIFC2	kinesin family member C2	-0.765120676	0.048111872	-1
8349	HIST2H2BE	histone cluster 2 H2B family member e	-0.767334996	0.009245768	-1
4753	NELL2	neural EGFL like 2	-0.769619242	0.044167355	-1
1488	CTBP2	C-terminal binding protein 2	-0.770900949	0.00913684	-1
9069	CLDN12	claudin 12	-0.770949514	0.009120082	-1
9858	PPP1R26	protein phosphatase 1 regulatory subunit 26	-0.771446509	0.009104682	-1
2983	GUCY1B3	guanylate cyclase 1 soluble subunit beta	-0.773291997	0.040420087	-1
154	ADRB2	adrenoceptor beta 2	-0.775414871	0.009000736	-1
78987	CRELD1	cysteine rich with EGF like domains 1	-0.787592063	0.048026949	-1
80833	APOL3	apolipoprotein L3	-0.789075712	0.008489385	-1
84264	HAGHL	hydroxyacylglutathione hydrolase-like	-0.790303461	0.022042235	-1
51191	HERC5	HECT and RLD domain containing E3 ubiquitin protein ligase 5	-0.792473137	0.015372247	-1
158248	TTC16	tetratricopeptide repeat domain 16	-0.795308648	0.008301421	-1
83959	SLC4A11	solute carrier family 4 member 11	-0.796102392	0.008255223	-1
8460	TPST1	tyrosylprotein sulfotransferase 1	-0.797033216	0.008215139	-1
4758	NEU1	neuraminidase 1	-0.803067662	0.008103493	-1
6351	CCL4	C-C motif chemokine ligand 4	-0.804101441	0.008037366	-1
84981	MIR22HG	MIR22 host gene	-0.806208027	0.026383966	-1
1534	CYB561	cytochrome b561	-0.806294235	0.007938176	-1
5291	PIK3CB	phosphatidylinositol-4,5-bisphosphate 3-kinase catalytic subunit beta	-0.812551342	0.007751345	-1
2999	GZMH	granzyme H	-0.815117708	0.03948661	-1
57103	TIGAR	TP53 induced glycolysis regulatory phosphatase	-0.817976141	0.025724509	-1
116039	OSR2	odd-skipped related transciption factor 2	-0.819059078	0.007580139	-1
2529	FUT7	fucosyltransferase 7	-0.823390768	0.00748661	-1
924	CD7	CD7 molecule	-0.824081128	0.007479364	-1

87	ACTN1	actinin alpha 1	-0.824333172	0.04143577	-1
10875	FGL2	fibrinogen like 2	-0.829324779	0.036138368	-1
467	ATF3	activating transcription factor 3	-0.832932738	0.031919832	-1
28962	OSTM1	osteopetrosis associated transmembrane protein 1	-0.837552163	0.019939082	-1
388228	SBK1	SH3 domain binding kinase 1	-0.838587356	0.0475353	-1
3490	IGFBP7	insulin like growth factor binding protein 7	-0.841456565	0.007116118	-1
25798	BRI3	brain protein I3	-0.843212708	0.007074902	-1
26115	TANC2	tetratricopeptide repeat, ankyrin repeat and coiled-coil containing 2	-0.846468981	0.026041782	-1
5734	PTGER4	prostaglandin E receptor 4	-0.848086726	0.006924532	-1
55652	SLC48A1	solute carrier family 48 member 1	-0.851081447	0.006866557	-1
9620	CELSR1	cadherin EGF LAG seven-pass G-type receptor 1	-0.860625616	0.006699655	-1
80705	TSGA10	testis specific 10	-0.875049413	0.008888411	-1
80328	ULBP2	UL16 binding protein 2	-0.876219364	0.033515937	-1
89884	LHX4	LIM homeobox 4	-0.87674284	0.006312178	-1
387856	CCDC184	coiled-coil domain containing 184	-0.87727207	0.033276793	-1
94134	ARHGAP12	Rho GTPase activating protein 12	-0.878251711	0.006279115	-1
26524	LATS2	large tumor suppressor kinase 2	-0.885875817	0.006133726	-1
374383	NCR3LG1	natural killer cell cytotoxicity receptor 3 ligand 1	-0.888678179	0.006048576	-1
94241	TP53INP1	tumor protein p53 inducible nuclear protein 1	-0.890345906	0.015681141	-1
79658	ARHGAP10	Rho GTPase activating protein 10	-0.89351214	0.005966597	-1
375387	NRROS	negative regulator of reactive oxygen species	-0.894601443	0.005949839	-1
8787	RGS9	regulator of G-protein signaling 9	-0.904014487	0.005763687	-1
124093	CCDC78	coiled-coil domain containing 78	-0.904779194	0.013449357	-1
55450	CAMK2N1	calcium/calmodulin dependent protein kinase II inhibitor 1	-0.907893425	0.005703221	-1
2841	GPR18	G protein-coupled receptor 18	-0.926020549	0.005358773	-1
7227	TRPS1	transcriptional repressor GATA binding 1	-0.93319616	0.026948084	-1
6622	SNCA	synuclein alpha	-0.938633281	0.021321633	-1
9214	FCMR	Fc fragment of IgM receptor	-0.94427654	0.033345638	-1
5820	PVT1	Pvt1 oncogene (non-protein coding)	-0.949590513	0.005023835	-1
23413	NCS1	neuronal calcium sensor 1	-0.953495096	0.047528053	-1
27113	BBC3	BCL2 binding component 3	-0.954226098	0.009986752	-1
101930370	LOC101930370	uncharacterized LOC101930370	-0.954744029	0.023758818	-1
10252	SPRY1	sprouty RTK signaling antagonist 1	-0.960667329	0.004802355	-1
57616	TSHZ3	teashirt zinc finger homeobox 3	-0.966872737	0.049726094	-1
9572	NR1D1	nuclear receptor subfamily 1 group D member 1	-0.973829875	0.006753326	-1
9764	KIAA0513	KIAA0513	-0.976398878	0.048832475	-1
54	ACP5	acid phosphatase 5, tartrate resistant	-0.984454715	0.006917285	-1
197135	PATL2	PAT1 homolog 2	-0.98658509	0.025802185	-1
100507421	TMEM178B	transmembrane protein 178B	-0.987686591	0.004497084	-1
1847	DUSP5	dual specificity phosphatase 5	-0.989606342	0.004473532	-1
6300	MAPK12	mitogen-activated protein kinase 12	-0.990112405	0.010550869	-1
970	CD70	CD70 molecule	-0.993945864	0.03009953	-1
952	CD38	CD38 molecule	-1.009606906	0.004158297	-1
4646	MYO6	myosin VI	-1.012325458	0.030222725	-1
2232	FDXR	ferredoxin reductase	-1.017557958	0.009537904	-1
23654	PLXNB2	plexin B2	-1.021715899	0.041509823	-1
2982	GUCY1A3	guanylate cyclase 1 soluble subunit alpha	-1.027587623	0.009967503	-1
644815	FAM83G	family with sequence similarity 83 member G	-1.028866996	0.04686384	-1
5521	PPP2R2B	protein phosphatase 2 regulatory subunit Bbeta	-1.029652113	0.009044896	-1
64283	ARHGEF28	Rho guanine nucleotide exchange factor 28	-1.041086214	0.003723943	-1
102724571	LINC01759	long intergenic non-protein coding RNA 1759	-1.047938169	0.003651248	-1
64651	CSRNP1	cysteine and serine rich nuclear protein 1	-1.053288972	0.022578271	-1
338596	ST8SIA6	ST8 alpha-N-acetyl-neuraminide alpha-2,8-sialyltransferase 6	-1.078760542	0.041099021	-1
634	CEACAM1	carcinoembryonic antigen related cell adhesion molecule 1	-1.082020915	0.027292759	-1
8061	FOSL1	FOS like 1, AP-1 transcription factor subunit	-1.09430614	0.003238634	-1
1647	GADD45A	growth arrest and DNA damage inducible alpha	-1.094551195	0.003231388	-1
26056	RAB11FIP5	RAB11 family interacting protein 5	-1.105461626	0.034789107	-1
57161	PELI2	pellino E3 ubiquitin protein ligase family member 2	-1.109507444	0.003074676	-1
4065	LY75	lymphocyte antigen 75	-1.118878806	0.002972994	-1
57480	PLEKHG1	pleckstrin homology and RhoGEF domain containing G1	-1.12542017	0.004185246	-1
8722	CTSF	cathepsin F	-1.128168248	0.008283983	-1
64393	ZMAT3	zinc finger matrin-type 3	-1.13475209	0.013167412	-1
23645	PPP1R15A	protein phosphatase 1 regulatory subunit 15A	-1.139398846	0.016195437	-1
8797	TNFRSF10A	TNF receptor superfamily member 10a	-1.141307173	0.04522131	-1
378805	LINC-PINT	long intergenic non-protein coding RNA, p53 induced transcript	-1.148598539	0.009852233	-1
8353	HIST1H3E	histone cluster 1 H3 family member e	-1.150114905	0.002720263	-1
8277	TKTL1	transketolase like 1	-1.153061394	0.04827651	-1
56172	ANKH	ANKH inorganic pyrophosphate transport regulator	-1.163231503	0.002614731	-1
9560	CCL4L2	C-C motif chemokine ligand 4 like 2	-1.179584793	0.020748004	-1
132671	SPATA18	spermatogenesis associated 18	-1.181977624	0.005412218	-1
59	ACTA2	actin, alpha 2, smooth muscle, aorta	-1.200231825	0.024798732	-1
10346	TRIM22	tripartite motif containing 22	-1.200480947	0.01338776	-1
55924	FAM212B	family with sequence similarity 212 member B	-1.202952146	0.006126479	-1
1612	DAPK1	death associated protein kinase 1	-1.210902634	0.023003114	-1
9697	TRAM2	translocation associated membrane protein 2	-1.220254216	0.029147031	-1
944	TNFSF8	tumor necrosis factor superfamily member 8	-1.223919192	0.002393704	-1
26049	FAM169A	family with sequence similarity 169 member A	-1.225749506	0.002229293	-1

133746	JMY	junction mediating and regulatory protein, p53 cofactor	-1.230247085	0.002345921	-1
220001	VWCE	von Willebrand factor C and EGF domains	-1.23903877	0.048431863	-1
2354	FOSB	FosB proto-oncogene, AP-1 transcription factor subunit	-1.242829235	0.032906301	-1
29940	DSE	dermatan sulfate epimerase	-1.246039684	0.020542377	-1
10014	HDAC5	histone deacetylase 5	-1.248359105	0.002076657	-1
8795	TNFRSF10B	TNF receptor superfamily member 10b	-1.264742703	0.001989017	-1
22821	RASA3	RAS p21 protein activator 3	-1.267524595	0.027255619	-1
57568	SIPA1L2	signal induced proliferation associated 1 like 2	-1.271166656	0.01088558	-1
27065	NSG1	neuron specific gene family member 1	-1.27403981	0.033313707	-1
55790	CSGALNACT1	chondroitin sulfate N-acetylgalactosaminyltransferase 1	-1.282850066	0.001904999	-1
8644	AKR1C3	aldo-keto reductase family 1 member C3	-1.285634775	0.001887562	-1
23467	NPTXR	neuronal pentraxin receptor	-1.288193224	0.002661156	-1
340527	NHSL2	NHS like 2	-1.291531482	0.001839778	-1
23209	MLC1	megalencephalic leukoencephalopathy with subcortical cysts 1	-1.307915135	0.003704693	-1
10769	PLK2	polo like kinase 2	-1.322030089	0.02653547	-1
6402	SELL	selectin L	-1.330759483	0.049418559	-1
79836	LONRF3	LON peptidase N-terminal domain and ring finger 3	-1.34314206	0.03068267	-1
106614088	MIR34AHG	MIR34A host gene	-1.349534612	0.007414822	-1
11126	CD160	CD160 molecule	-1.397170044	0.015365	-1
23612	PHLDA3	pleckstrin homology like domain family A member 3	-1.45876761	0.00129174	-1
9429	ABCG2	ATP binding cassette subfamily G member 2 (Junior blood group)	-1.480140541	0.001230142	-1
56654	NPDC1	neural proliferation, differentiation and control 1	-1.504864228	0.001142048	-1
23108	RAP1GAP2	RAP1 GTPase activating protein 2	-1.509400184	0.006339806	-1
10577	NPC2	NPC intracellular cholesterol transporter 2	-1.515932506	0.009681934	-1
6448	SGSH	N-sulfoglucosamine sulfohydrolase	-1.5330715	0.001109438	-1
4248	MGAT3	mannosyl (beta-1,4-)-glycoprotein beta-1,4-N-acetylglucosaminyltransferase	-1.559505065	0.011437695	-1
222166	MTURN	maturin, neural progenitor differentiation regulator homolog	-1.562780512	0.001043311	-1
10365	KLF2	Kruppel like factor 2	-1.610998959	0.030332786	-1
25841	ABTB2	ankyrin repeat and BTB domain containing 2	-1.68196009	0.000806658	-1
54796	BNC2	basonuclin 2	-1.689588304	0.01480496	-1
1524	CX3CR1	C-X3-C motif chemokine receptor 1	-1.713255545	0.000766574	-1
8793	TNFRSF10D	TNF receptor superfamily member 10d	-1.745300922	0.002147087	-1
5973	RENBP	renin binding protein	-1.754028268	0.029566212	-1
10253	SPRY2	sprouty RTK signaling antagonist 2	-1.781832069	0.000642473	-1
7292	TNFSF4	tumor necrosis factor superfamily member 4	-1.992769287	0.000439563	-1
134147	CMBL	carboxymethylenebutenolidase homolog	-1.995368355	0.000432316	-1
83888	FGFBP2	fibroblast growth factor binding protein 2	-2.053898654	0.006692408	-1
284129	SLC26A11	solute carrier family 26 member 11	-2.102825487	0.000512937	-1
